# Cyclomulberrin from *Morus alba* L. exerts antithrombotic effects by modulating platelet activation: an integrated network pharmacology and *in vitro* study

**DOI:** 10.1039/d6ra00546b

**Published:** 2026-07-02

**Authors:** Yanqiong Guo, Rongrong He, Jing Peng, Shuguo Yuan, Yanlei Ma, Qingde Li

**Affiliations:** a Department of Pharmacy, Yuebei People's Hospital, Shantou University Medical College Shaoguan 512026 China gyqiong@mail2.sysu.edu.cn; b Department of Cardiology, Yuebei People's Hospital, Shantou University Medical College Shaoguan 512026 China 419090549@qq.com

## Abstract

Despite the documented bioactivity of *Morus alba* L., the antithrombotic mechanism of its constituent flavonoid cyclomulberrin is not fully understood. Here, we applied network pharmacology, molecular docking/dynamics, and *in vitro* assays to evaluate its antithrombotic properties. The compound was isolated from Sang-Bai-Pi (root bark of *M. alba* L). Network pharmacology identified 57 potential targets and seven hub targets (TNF, AKT1, SRC, PTGS2, MMP9, PPARG, CYCS) enriched in platelet activation, PI3K-Akt, cAMP, and TNF pathways. Molecular docking and 100-ns molecular dynamics simulations confirmed stable binding to all seven hubs. Selective inhibitor experiments (PP2, MK-2206) confirmed functional engagement of SRC and AKT1. Cyclomulberrin potently inhibited key platelet responses: aggregation, ATP release, αIIbβ3 activation, Ca^2+^ mobilization, and ROS generation, while elevating cAMP. Coagulation parameters were unaffected below 40 µM. Our results demonstrate that cyclomulberrin engages multiple targets *via* a multi-pathway mechanism, positioning it as a promising natural-source antithrombotic agent.

## Abstract 2

Network pharmacology, docking/MD, and platelet assays reveal that cyclomulberrin from *Morus alba* modulates platelet activation *via* multi-target/multi-pathway mechanisms, conferring antithrombotic effects without affecting coagulation.

## Graphical abstract

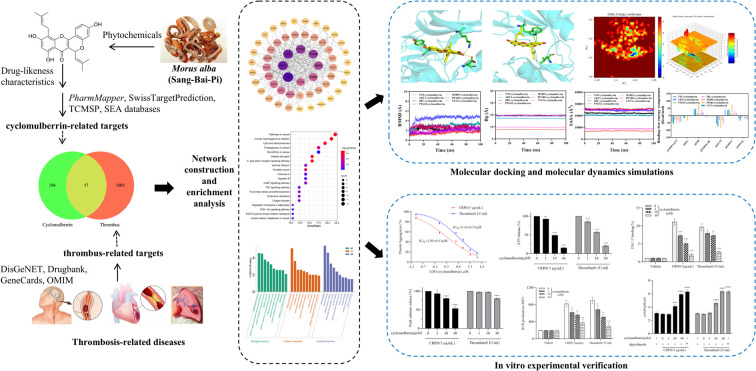

## 1. Introduction

Thrombosis-related diseases, especifically ischemic heart disease, ischemic stroke, and venous thromboembolism, are major contributors to global mortality and account for a significant portion of the worldwide disease burden as reflected by disability-adjusted life years.^1–3^ The most prevalent pathogenesis of these diseases is thrombosis, a multifaceted and complex biochemical process characterized by platelet activation, aggregation, and fibrin deposition.^4–6^ Given the pivotal role platelets play in thrombus formation, they emerge as a crucial therapeutic target in the prevention and treatment of thrombotic disorders. A series of antiplatelet drugs such as aspirin and clopidogrel have been used clinically in the management of thrombotic diseases. However, the currently available antiplatelet drugs are frequently linked with adverse effects ranging from gastrointestinal toxicity^7^ and gastric bleeding^8^ to severe intracranial hemorrhage.^9^ Additionally, some patients exhibit tolerance to these treatments.^10,11^ Thus, there is a pressing clinical demand for the development of safer and more efficacious antithrombotic agents.^12^

Traditional Chinese medicine (TCM) is recognized for its good curative effect, minimal side effects, and low cost in treating diseases through multiple targets, pathways and links.^13^ This has aroused great interest in developing new antithrombotic drugs derived from TCM.^14,15^ Sang-Bai-Pi (a TCM in Chinese), the root bark of *Morus alba* L (*Moraceae*), has been traditionally used to treat respiratory disorders like cough, asthma, bronchitis and pulmonary diseases, as well as hepatitis, diabetes, inflammation, and hypertension.^16–18^ Flavonoids, the primary bioactive components of *Morus alba*, have been associated with various pharmacological effects such as anti-inflammatory responses, free radical scavenging, antimicrobial activities, and antitumor properties.^18,19^ Recent studies have highlighted the potential of *Morus alba* in preventing and treating thrombosis, as the naturally derived ingredients including Morusinol, Morin hydrate, and Mulberroside have been demonstrated to suppress platelet activation, and some of these ingredients have been shown to diminish arterial or venous thrombosis in animal models.^20–23^ Our previous studies have identified certain flavonoids from *Morus alba*, including cyclomulberrin, as potent inhibitors of phosphodiesterase-4,^24^ an enzyme that plays a crucial role in cAMP hydrolysis and myocardial contraction.^25^ However, despite the comprehensive study of *Morus alba*'s chemical composition and pharmacological activities, the specific role of cyclomulberrin on platelet function and thrombosis remains poorly understood.

Network pharmacology is an effective approach for establishing “compound-protein/gene-disease” networks that reveal the regulatory mechanisms of small molecule compounds.^26–28^ In this study, we applied network pharmacology to explore the therapeutic potential and mechanism of cyclomulberrin in thrombotic diseases. Furthermore, we conducted molecular docking, molecular dynamics simulations and *in vitro* experiments covering platelet aggregation, granule release, αIIbβ3 activation, calcium mobilization, cAMP levels, oxidative stress, and blood coagulation to validate cyclomulberrin's function in thrombotic diseases.

## 2. Materials and methods

### 2.1. Extraction and isolation of cyclomulberrin from *Morus alba*

The root bark of *Morus alba* (1.5 kg), also known as Sang-Bai-Pi in Chinese, was air-dried, then crushed to powder and extracted with 95% ethanol (EtOH) at room temperature to obtain a crude extract. The extract was later suspended in water and partitioned sequentially with petroleum ether, ethyl acetate (EtOAc), and *n*-BuOH. Further purification through various column chromatographic processes on the EtOAc extract yielded cyclomulberrin (400 mg, detailed procedures are provided in the SI). The purity of cyclomulberrin was confirmed to exceed 95% using ^1^H- and ^13^C- NMR spectra.

### 2.2. Evaluation of drug-likeness for cyclomulberrin

The 2D and 3D structural SDF formats and canonical SMILES of cyclomulberrin were retrieved from the PubChem database (https://pubchem.ncbi.nlm.nih.gov/). The drug-likeness for oral drugs in humans is typically evaluated using Lipinski's Rule of Five (RO5). This rule takes into consideration parameters like molecular weight (*M*_W_, < 500 g mol^−1^), topological polar surface area (TPSA, < 140 A^2^), octanol–water partition coefficient (log *P*_o/W_ (MLOGP), < 5), number of hydrogen-bond acceptors (*n*HAcc, < 10), number of hydrogen-bond donors (*n*HDon, < 5), and number of rotatable bonds (<10). To estimate the drug-like and other properties of cyclomulberrin, we loaded its canonical SMILES CC(=CCC1=C2C(=C(C=C1O)O)C(=O)C3=C(O2)C4=C(C=C(C=C4)O)OC3C=C(C)C)C into the SwissADME database (https://www.swissadme.ch/).

### 2.3. Network pharmacology

#### 2.3.1. Identification of cyclomulberrin- and thrombus-related target

The pharmacological targets of cyclomulberrin were identified by using the *PharmMapper* (https://www.lilab-ecust.cn/pharmmap), SwissTargetPrediction (https://www.swisstargetprediction.ch/), TCMSP (https://old.tcmsp-e.com/tcmsp.php), and SEA (https://sea.bkslab.org/) databases. Separately, thrombus-related targets were selected from the DisGeNET (https://www.disgenet.org/search), Drugbank (https://go.drugbank.com/), GeneCards (https://www.genecards.org/) and OMIM (https://www.omim.org) databases. The overlapping target genes for both cyclomulberrin and thrombus were identified using Venny 2.1 (https://bioinfogp.cnb.csic.es/tools/venny/), and these common genes were then postulated as cyclomulberrin's potential antithrombotic targets.

#### 2.3.2. PPI network and hub targets analysis

Utilizing the STRING database (https://www.string-db.org/), we constructed a PPI network which was further visualized with Cytoscape software (version 3.9.1). To identify the hub targets, we applied the CytoHubba plug-in of Cytoscape to calculate the score of each node and rank the top 10 hub targets based on degree, stress, betweenness, MCC (Maximal Clique Centrality), and EPC (Edge Percolated Component).

#### 2.3.3. GO and KEGG pathway enrichment analysis

GO and KEGG pathway enrichments were performed *via* the DAVID software (https://david-d.ncifcrf.gov/). Statistically significant enrichment results (adjusted *p*-value) were selected and then visually represented using bar graphs and bubble plots (https://www.bioinformatics.com.cn). The subsequent compound/disease-target-pathway network was constructed using Cytoscape software.

### 2.4. Molecular docking and molecular dynamics simulations

#### 2.4.1. Molecular docking and redocking validation

We used AutoDock Vina 1.5.6 (https://autodock.scripps.edu/)^29^ to dock cyclomulberrin into the previously identified hub targets. Crystal structures (resolution ≤2.5 Å, with native ligand and intact binding pocket) were downloaded from the PDB (https://www.rcsb.org/):^30^ TNF (2AZ5, 2.10 Å), AKT1 (8Q61, 2.32 Å), SRC (2H8H, 2.20 Å), PTGS2 (3LN1, 2.40 Å), MMP9 (6ESM, 1.10 Å), PPARG (8B92, 1.66 Å), and CYCS (5TY3, 1.25 Å). Water molecules, phosphate ions, and other heteroatoms were removed with PyMOL. Polar hydrogens were added, and proteins and ligand were converted to PDBQT format with atomic charges and torsional degrees of freedom. The grid box (47.25 Å × 47.25 Å × 47.25 Å) was centered on the original co-crystallized ligand coordinates to cover the entire binding pocket. For validation, we extracted and re-docked the native co-crystallized ligand using the same parameters. The RMSD between the re-docked pose and the original crystal pose was calculated; an RMSD < 2.0 Å was considered acceptable. For each target we selected the pose with the lowest binding affinity. Results were visualized with Discovery Studio 2019 and PyMOL.

#### 2.4.2. Molecular dynamics simulations

The cyclomulberrin – target complexes from docking were further simulated by molecular dynamics (MD) using GROMACS software (version 2022).^31^ Proteins were modeled with the AMBER14SB force field. Ligand topologies were generated with Sobtop (GAFF2 force field),^32^ and partial atomic charges were assigned *via* RESP.^33^ Each complex was placed in a dodecahedron box with a 1.0 nm margin, solvated with TIP3P water,^34^ and neutralized with Na^+^/Cl^−^ to 0.15 M ionic strength. Long-range electrostatics used particle mesh Ewald (PME) with a 1.0 nm cutoff; all bonds were constrained with LINCS. Energy minimization: 3000 steps steepest descent, then 2000 steps conjugate gradient until max force <1000 kJ (mol nm)^−1^. The system was equilibrated under NVT (100 ps, 310 K, Nosé–Hoover) then NPT (100 ps, 1 bar, Parrinello–Rahman).^35^ Production MD ran for 100 ns (2 fs time step), saving trajectories every 10 ps. Trajectories were analyzed with built-in GROMACS tools for root-mean-square deviation (RMSD), root-mean-square fluctuation (RMSF), radius of gyration (Rg), solvent accessible surface area (SASA), Hydrogen Bond (Hbond), and free energy landscape (FEL, *via* gmx sham). Binding free energies were calculated by the gmx_MMPBSA tool (MM/PBSA method)^36^ using the last 10 ns as the equilibrium phase. Per-residue energy decomposition then identified key interacting residues.

### 2.5. *In Vitro* experiments

#### 2.5.1. Platelet preparation

Blood samples were sourced from healthy individuals, collected into tubes containing the anticoagulant mix of trisodium citrate, glucose, and citric acid (ACD). The samples were then centrifuged at 120×*g* for 20 minutes at room temperature (25 °C) to obtain the platelet-rich plasma (PRP). The PRP was further centrifuged at 1350×*g* for 15 minutes to isolate the platelet pellets. These were then washed thrice with CGS buffer and finally resuspended in Tyrode's buffer, adjusting the platelet suspension concentration to 5 × 10^8^ mL^−1^. All experiments involving human blood were performed in accordance with the Declaration of Helsinki, and were approved by the Medical Ethics Committee of Yuebei People's Hospital (approval no. KY-2021-221). Informed consents were obtained from human participants of this study. For subsequent experiments, the platelet concentration was adjusted to 1 × 10^8^ mL^−1^ with Tyrode's buffer.

#### 2.5.2. Platelet aggregation assay

Platelets (1 × 10^8^ mL^−1^, prepared as in 2.5.1) were incubated with various concentrations of cyclomulberrin (0.1–40 µM) or a control for 5 minutes at 37 °C. All incubations and reactions were carried out at 37 °C unless otherwise stated. Post-incubation, the samples were stimulated with 0.1U ml^−1^ thrombin, 0.5 µg ml^−1^ CRP, 2.5 µM ADP, or 5.0 µM U46619. Platelet aggregation was then evaluated by monitoring light transmission with an aggregometer (Chrono-Log, USA) stirred at 1000 rpm. The platelet aggregation levels were expressed percentages, benchmarked against the maximum platelet aggregation observed in the cyclomulberrin-free sample. Based on the aggregation results, thrombin and CRP were used as the agonists in all subsequent assays.

#### 2.5.3. Cytotoxicity assay

The lactate dehydrogenase (LDH) cytotoxicity assay was carried out using the provided LDH assay kit (Beyotime, China). Platelets were treated with different concentrations of cyclomulberrin, followed by addition of the reaction mixture from the kit and incubation for 30 minutes. Then, a stop solution halted the reaction, and the absorbance of the mixture was measured with a spectrophotometer (PerkinElmer, USA).

#### 2.5.4. Measurement of ATP release

To determine platelet dense granule secretion, ATP release was measured using the luciferin-luciferase reagent through aggregometry. Specifically, platelets underwent incubation with the Chrono-Lume reagent for 5 minutes. This was followed by exposure to various concentrations of cyclomulberrin for another 5 minutes, before being activated with the aforementioned agonists. The ATP release was then quantified and compared to a vehicle control.

#### 2.5.5. Measurement of platelet α-granule secretion and αIIbβ3 activation

Platelet α-granule secretion was assessed by monitoring the P-selectin expression. Additionally, the activation-dependent binding of PAC-1 to platelet αIIbβ3 served as a metric to gauge the activation of integrin αIIbβ3. After cyclomulberrin treatment and agonists stimulation, platelets were incubated with either PE-conjugated anti-P-selectin antibody or FITC-conjugated PAC-1 antibody for 15 minutes. The resultant P-selectin expression or αIIbβ3 activation in the activated platelets was detected using flow cytometry (Beckman, USA).

#### 2.5.6. Calcium mobilization

The intracellular calcium levels in platelets were assessed using Fluo-4/AM (Invitrogen, China) in accordance with the manufacturer's protocol. Platelets, loaded with Fluo-4/AM after a 45-minute incubation at 30 °C in darkness, were then exposed to either a control vehicle or various concentrations of cyclomulberrin for 5 minutes. Subsequent activation with agonists was followed by measuring light emissions at 520 nm (excited at 480 nm) *via* a fluorescence spectrophotometer (PerkinElmer, USA). The relative change in calcium concentration was computed by comparing it to the maximum observed calcium release across samples.

#### 2.5.7. Measurement of ROS in platelets

Utilizing the 2′, 7′-dichlorodihydrofluorescein diacetate (H2DCF-DA; Sigma-Aldrich, USA) and flow cytometry, intracellular H_2_O_2_ levels in platelets were quantified.^37^ After exposure to either the vehicle control or cyclomulberrin, platelets were loaded with H2DCF-DA and incubated for 30 minutes in a light-free setting. Following stimulation with agonists, ROS generation triggered fluorescence, which was captured using flow cytometry. Fluorescence intensity was expressed as mean fluorescence intensity (MFI).

#### 2.5.8. Measurement of cAMP production

After conducting the platelet aggregation assay as described above, ice-cold 80% ethanol was added and vortexing was carried out, the samples were allowed to rest for 5 minutes at room temperature and then centrifuged at 2000×*g* and 4 °C for 10 minutes. The cAMP levels in the samples were determined utilizing the cAMP ELISA kit (Cayman Chemical, USA) under the manufacturer's recommended protocol.

#### 2.5.9. Functional target engagement validation using selective inhibitors

To test whether SRC and AKT1 are functionally involved, we used cellular functional target engagement assay with selective inhibitors.^38^ Platelets were pre-incubated for 30 minutes with either the selective SRC family kinase (SFKs) inhibitor PP2 ^39^ (10 µM, GlpBio, CAS 172889-27-9) or the selective pan-AKT inhibitor MK-2206 ^40^ (1 µM, Adooq, CAS 1032350-13-2). DMSO (≤0.1%, v/v) was used as vehicle control. After pre-incubation, platelets were treated with cyclomulberrin (5 µM) or DMSO for 10 minutes, and then CRP was added to trigger aggregation. Groups: DMSO + CRP (control), cyclomulberrin + CRP, PP2+CRP, PP2+cyclomulberrin + CRP, MK-2206+CRP, and MK-2206+cyclomulberrin + CRP.

#### 2.5.10. PT and aPTT assay

Centrifugation of human PRP at 500×*g* for 10 minutes yielded platelet-poor plasma (PPP). These PPP samples were subsequently incubated with cyclomulberrin or a vehicle control for 5 minutes. To determine PT, thromboplastin-D reagent was added to the PPP; for aPTT measurement, CaCl_2_ and partial thromboplastin were added. The time to fibrin clot formation was recorded *via* a coagulometer (Ruimai, China).

### 2.6. Statistical analysis

Data obtained was expressed as the mean ± standard deviation (SD) and was analyzed using Graph Pad Prism 8.0.2 (Graph Pad Software Inc., USA) and SPSS 26.0 software (IBM Corporation, USA). Differences between groups were assessed using the *t*-test or one-way analysis of variance (ANOVA). All experiments were carried out at least three times, and a *p*-value of less than 0.05 was considered statistically significant.

## 3. Results

### 3.1. Molecular properties of cyclomulberrin

**Fig. 1:**
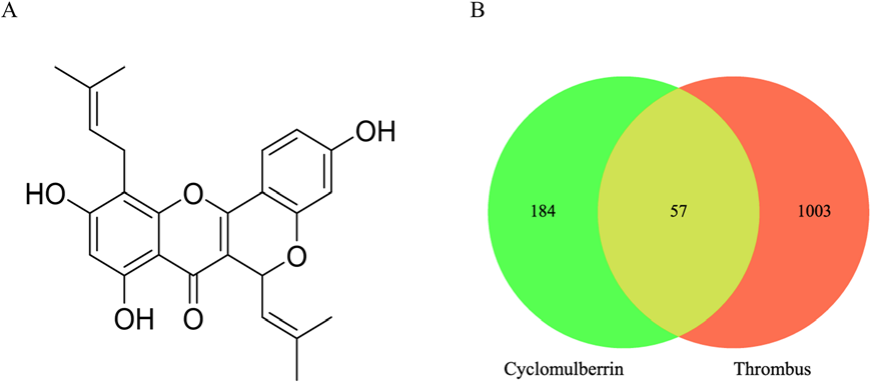
Chemical structure and Venn diagram. (A) Chemical structure of cyclomulberrin. (B) Venn diagram of cyclomulberrin- and thrombus-related targets.

From 1.5 kg of Sang-Bai-Pi, 400 mg of cyclomulberrin was acquired, and its NMR characterization was carried out as follows:1H-NMR (400 MHz, Acetone-d_6_) *δ* 7.69 (1H, d, *J* = 7.2 Hz, H-6′), 6.41 (1H, dd, *J* = 7.2, 2.2 Hz, H-5′), 6.43 (1H, s, H-6), 6.33 (1H, d, *J* = 2.2 Hz, H-3′), 6.18 (1H, d, *J* = 9.4 Hz, H-10), 5.47 (1H, d, *J* = 9.4 Hz, H-9), 5.31 (1H, t, *J* = 7.0 Hz, H-15), 3.53 (2H, m, H-14), 1.93 (3H, s, CH_3_-12), 1.83 (3H, s, CH_3_-13), 1.67 (3H, s, CH_3_-17), 1.65 (3H, s, CH_3_-18); 13C-NMR (100 MHz, Acetone-d_6_) *δ* 179.3.0 (C-4), 164.2 (C-4′), 162.8 (C-7), 160.9 (C-2), 159.0 (C-5), 156.4 (C-8a), 155.2 (C-2′), 131.9 (C-16), 107.5 (C-3), 126.1 (C-6′), 123.5 (C-15), 122.3 (C-10), 110.9 (C-5′), 109.6 (C-1′), 108.6 (C-8), 107.5 (C-3), 105.4 (C-4a), 104.9 (C-3′), 99.4 (C-6), 70.4 (C-9), 25.9 (C-12), 25.9 (C-17), 22.3 (C-14), 18.6 (C-18), 18.1 (C-13). The determined molecular structure of cyclomulberrin is depicted in Fig. 1A, with a molecular formula of C_25_H_24_O_6_. We examined 10 properties of cyclomulberrin and found that it had a MW of 420.45 g mol^−1^, *n*HAcc of 6, *n*HDon of 3, log *P*_O/W_ (MLOGP) of 2.09, rotatable bonds of 3, TPSA of 100.13 A^2^, log *S* (ESOL) of −6.32, high GI absorption, log *K*_p_ (skin permeation) of −4.71 cm s^−1^, and a bioavailability score of 0.55, as summarized in Table 1. Crucially, the molecular characteristics of cyclomulberrin are consistent with the Lipinski's Rule of Five (RO5), suggesting its potential as a drug candidate.

**Table 1:** Molecular properties of cyclomulberrin

Property	Value
Molecular weight	420.45 g mol^−1^
*n*HAcc	6
*n*HDon	3
Log *P*_O/W_(MLOGP)	2.09
Rotatable bonds	3
TPSA	100.13 A^2^
Log *S* (ESOL)	−6.23
Molar refractivity	121
Log *K*_p_ (skin permeation)	−4.71 cm s^−1^
Bioavailability score	0.55

### 3.2. Network pharmacology analysis

#### 3.2.1. Targets of cyclomulberrin and thrombus

A total of 241 targets (after removing duplicates) of cyclomulberrin were obtained through the SwissTargetPrediction, *PharmMapper*, TCMSP and SEA databases. Meanwhile, one thousand and sixty genes (after deleting duplicates) related to thrombus were identified from the DisGeNET, Drugbank, GeneCards, and OMIM databases. As shown in Fig. 1B utilizing Venn diagrams, there were 57 common targets as prospective targets of cyclomulberrin against thrombosis.

#### 3.2.2. Protein–protein interaction (PPI) network and hub targets

**Fig. 2:**
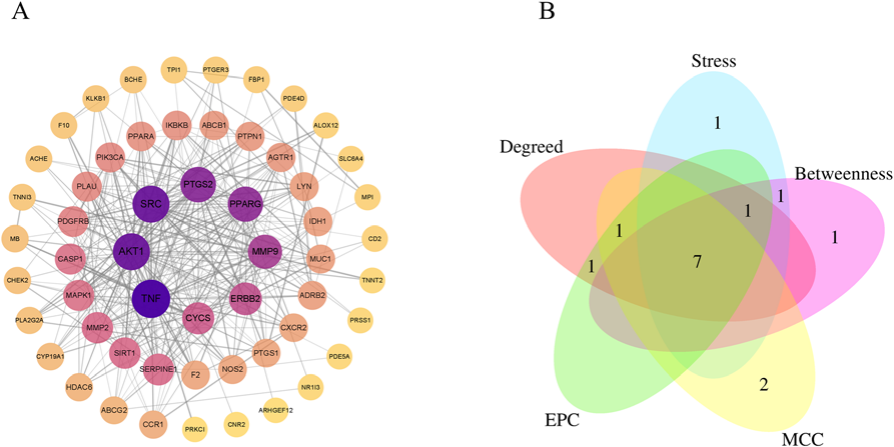
PPI network and hub targets analysis. (A) PPI network diagram. (B) Hub targets by topological analysis of CytoHubba.

To further explore the association between the 57 common target genes, we constructed a PPI network with 56 nodes and 312 edges using the STRING database (Fig. 2A). Furthermore, based on the CytoHubba topological analysis (Fig. 2B), seven key targets – TNF, AKT1, SRC, PTGS2, MMP9, PPARG, and CYCS were identified as hub targets pivotal for cyclomulberrin's antithrombotic effect (Table 2).

**Table 2:** Hub targets for cyclomulberrin in thrombosis treatment: binding affinity and redocking RMSD

Target	UnitProt ID	Description	PDB ID	Ligand	Binding affinity/(kcal mol^−1^)	Redocking RMSD (Å)
TNF	P01375	Tumor necrosis factor	2AZ5	Cyclomulberrin	−9.1	1.6
AKT1	P31749	RAC-alpha serine/threonine-protein kinase	8Q61	Cyclomulberrin	−10.4	1.07
SRC	P12931	Proto-oncogene tyrosine-protein kinase Src	2H8H	Cyclomulberrin	−9.7	0.25
PTGS2	P35354	Prostaglandin G/H synthase 2	3LN1	Cyclomulberrin	−6.4	0.01
MMP9	P14780	Matrix metalloproteinase-9	6ESM	Cyclomulberrin	−8.8	0.21
PPARG	P37231	Peroxisome proliferator-activated receptor gamma	8B92	Cyclomulberrin	−8.6	1.56
CYCS	P99999	Cytochrome c	5TY3	Cyclomulberrin	−7	0.01

#### 3.2.3. Gene ontology (GO) and KEGG pathway enrichment analysis

**Fig. 3:**
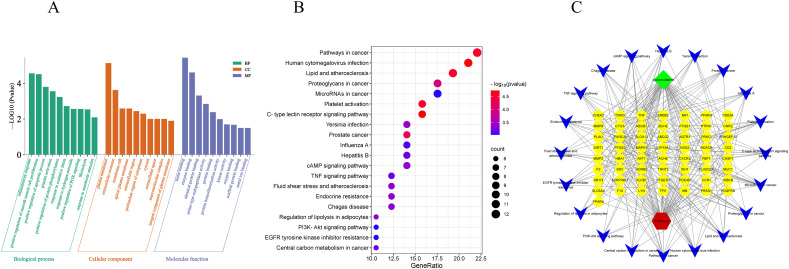
Enrichment analysis and Networks. GO (A) and KEGG pathway (B) enrichment analysis of common targets. (C) Compound/Disease-target-pathway networks (C/D-T-P).

The 57 common target genes were grouped into three categories based on GO classification: “biological processes” (BP), “cellular components” (CC) and “molecular functions” (MF). As shown in Fig. 3A, the top 10 BP terms analysis revealed that the majority of these targets are closely related to the inflammatory response, platelet activation, response to oxidative stress, response to hydrogen peroxide, and fibrinolysis. CC analysis indicated that these targets were primarily distributed in the plasma membrane, extracellular, cytosol, perinuclear region, and macromolecular complex. In terms of MF analysis, these targets were associated with the heme binding, enzyme binding, identical protein binding, protein binding, kinase activity, and oxygen binding (Fig. 3A). Collectively, these results implicate the targets of cyclomulberrin in key processes of thrombus formation.

Subsequent KEGG pathway enrichment analysis was performed to identify the pathways involving cyclomulberrin's antithrombotic targets. As shown in Fig. 3B, key pathways such as platelet activation, PI3K-Akt signaling, cAMP signaling, TNF signaling, and lipid and atherosclerosis were significantly enriched. A comprehensive multi-level interaction network (Fig. 3C) was constructed to provide clarity on the intricate relationships intertwining cyclomulberrin, thrombosis, targets and signaling pathways.

### 3.3. Molecular docking and molecular dynamics simulations

#### 3.3.1. Molecular docking and redocking validation

**Fig. 4:**
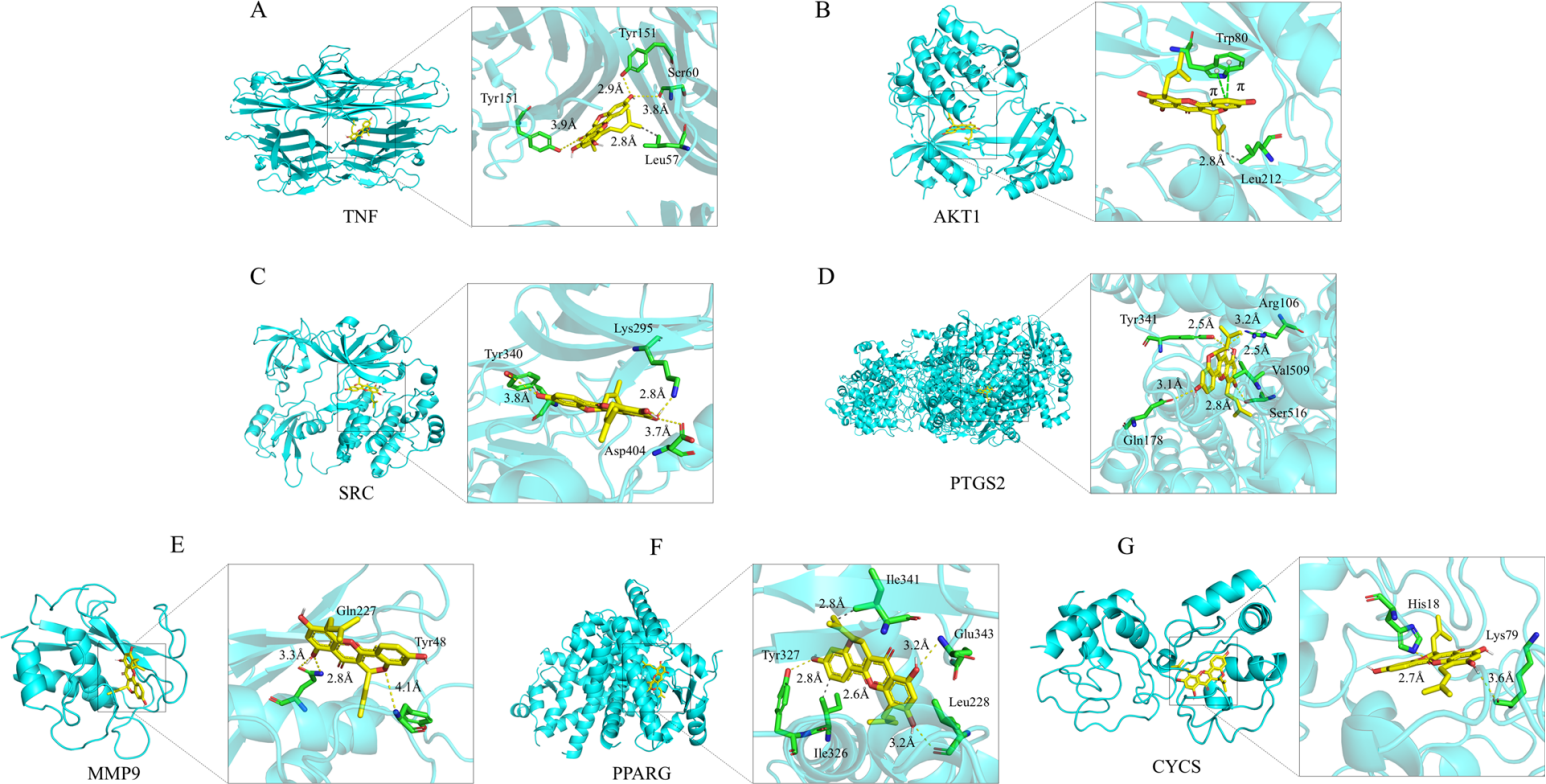
Binding modes and interactions of cyclomulberrin with the top seven hub targets. Binding mode and interactions of cyclomulberrin with TNF (A), AKT1 (B), SRC (C), PTGS2 (D), MMP9 (E), PPARG (F) and CYCS (G).

Cyclomulberrin was docked directly into the identified hub targets. Binding affinities (Table 2) were as follows: −9.10 (TNF), −10.40 (AKT1), −9.70 (SRC), −6.40 (PTGS2), −8.80 (MMP9), −8.60 (PPARG), and −7.00 kcal mol^−1^ (CYCS). Fig. 4 shows the key binding poses and interactions. In AKT1, cyclomulberrin had hydrophobic contacts with Leu212 and a π–π stack with Trp80. For SRC, three hydrogen bonds were established with the side chains of Lys295 (2.8 Å), Tyr340 (3.8 Å), and Asp404 (3.7 Å). For TNF, three H-bonds were seen: one with Ser60 (3.8 Å), and two with Tyr151 from different chains (2.9 Å and 3.9 Å); plus hydrophobic contacts with Leu57. In PPARG, cyclomulberrin formed hydrophobic interactions with Ile326 and Ile341; three H-bonds with Leu228 (3.2 Å), Tyr327 (2.8 Å), and Glu343 (3.2 Å). For PTGS2, four hydrogen bonds were observed with Arg106 (3.2 Å), Gln178 (3.1 Å), Tyr341 (2.5 Å), and Ser516 (3.2 Å), along with hydrophobic contacts with Val509. Redocking RMSD values were all below 2.0 Å (Table 2), confirming the docking parameters are reliable for these targets. Furthermore, cyclomulberrin and the native co-crystallized ligands occupied the same orthosteric pocket in each target (SI Fig. S8). Most interacting residues differed, but three residues appeared in common: Gln227 (MMP9), Leu228 (PPARG), and Lys295 (SRC) – suggesting some overlap in binding modes.

#### 3.3.2. Molecular dynamics simulations of cyclomulberrin – target complexes

**Fig. 5:**
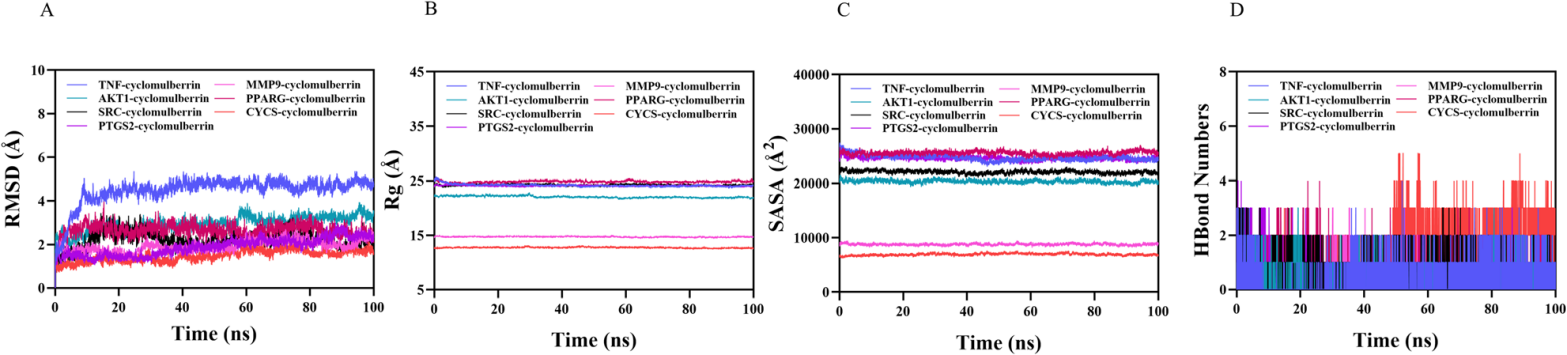
Molecular dynamics simulation of cyclomulberrin with seven hub target proteins. (A) RMSD, (B) *R*_g_, (C) SASA, (D) number of hydrogen bonds.

We ran 100 ns molecular dynamics (MD) simulations to check the stability of cyclomulberrin bound to the seven hub targets from network pharmacology and docking. RMSD was used to monitor complex stability. All systems equilibrated within 100 ns (Fig. 5A): TNF and PPARG stabilized by ∼10 ns, PTGS2 and SRC took ∼90 ns. Final RMSD values: TNF 1.54 Å, AKT1 3.50 Å, SRC 3.04 Å, PTGS2 2.72 Å, MMP9 2.30 Å, PPARG 2.74 Å, CYCS 1.84 Å – no significant conformational drift. Rg and SASA remained stable throughout (Fig. 5B–C), meaning no major conformational changes and little effect on hydrophilic/hydrophobic character, consistent with the RMSD analysis. Hydrogen bond analysis (Fig. 5D) revealed dynamic yet persistent interactions. Complexes with AKT1, MMP9, TNF, PTGS2 predominantly formed one hydrogen bond (range: 0–3 each), whereas SRC, PPARG, and CYCS frequently formed two (0–5). The MM/PBSA binding free energies (Table 3 and Fig. S11A) were all negative (kcal mol^−1^): TNF −25.40 ± 3.07, AKT1 −18.81 ± 3.58, SRC −26.50 ± 2.52, PTGS2 −22.68 ± 2.34, MMP9 −17.37 ± 3.58, PPARG −10.42 ± 4.18, CYCS −34.55 ± 3.66. More negative means stronger binding. Additional analyses (RMSF, free energy landscape, and per-residue energy decomposition) are provided in SI (Figs. S9–S11), further supporting the stability and highlighting key residues.

**Table 3:** Dynamic energy analysis of cyclomulberrin with seven hub target proteins^a^

Energy components	TNF-cyclomulberrin	AKT1-cyclomulberrin	SRC-cyclomulberrin	PTGS2-cyclomulberrin	MMP9-cyclomulberrin	PPARG-cyclomulberrin	CYCS-cyclomulberrin
ΔVDWAALS	−40.57 ± 3.57	−43.15 ± 2.42	−48.97 ± 3.20	−29.60 ± 3.22	−29.86 ± 3.33	−50.28 ± 3.44	−56.80 ± 3.01
ΔEEL	−1.65 ± 2.36	−12.12 ± 3.41	−8.99 ± 2.44	−5.59 ± 5.64	−0.48 ± 3.93	−33.11 ± 4.00	−18.00 ± 2.35
ΔEPB	23.01 ± 3.39	42.58 ± 4.45	37.22 ± 4.17	16.50 ± 4.75	17.09 ± 3.67	79.86 ± 5.56	46.64 ± 2.03
ΔENPOLAR	−6.19 ± 0.17	−6.11 ± 0.18	−5.76 ± 0.18	−3.99 ± 0.23	−4.11 ± 0.26	−6.90 ± 0.11	−6.38 ± 0.12
ΔGGAS	−42.22 ± 5.52	−55.27 ± 4.58	−57.96 ± 4.53	−35.19 ± 4.65	−30.34 ± 6.03	−83.39 ± 3.78	−74.80 ± 4.16
ΔGSOLV	16.83 ± 3.36	36.47 ± 4.35	31.47 ± 4.14	12.51 ± 4.77	12.98 ± 3.61	72.96 ± 5.60	40.26 ± 2.08
ΔTOTAL	−25.40 ± 3.07	−18.81 ± 3.58	−26.50 ± 2.52	−22.68 ± 2.34	−17.37 ± 3.58	−10.42 ± 4.18	−34.55 ± 3.66

a Values are were expressed as the mean ± standard deviation (kcal mol^−1^).

### 3.4. Experimental outcomes

#### 3.4.1. Cyclomulberrin inhibits platelet aggregation without cytotoxicity

**Fig. 6:**
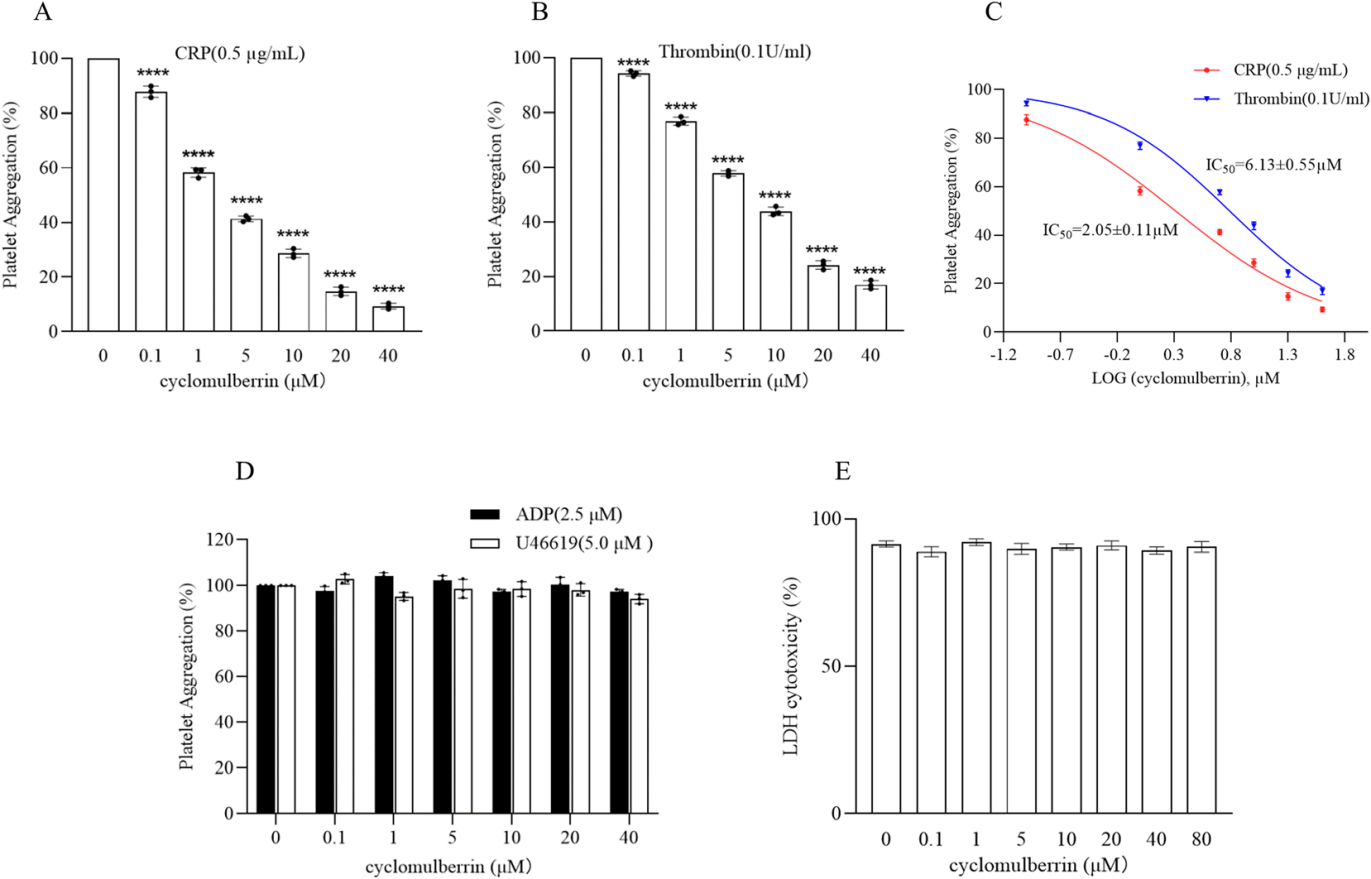
Effect of cyclomulberrin on platelet aggregation stimulated by CRP (A), thrombin (B), ADP and U46619 (C). (D) IC_50_ value of cyclomulberrin on CRP- and thrombin-induced platelet aggregation. (E) Cytotoxicity of cyclomulberrin on platelet.

We evaluated cyclomulberrin's impact on platelet activation through aggregation assays with various agonists. As shown in Fig. 6A and D, cyclomulberrin significantly inhibited CRP-induced platelet aggregation in a dose-dependent manner, with the 50% inhibitory concentration (IC_50_) being 2.05 ± 0.11 µM. In contrast, its capacity to inhibit thrombin-triggered platelet aggregation was weaker, with an IC_50_ of 6.13 ± 0.55 µM (Fig. 6B and D). Notably, such inhibitory effects were not observed under ADP and U46619 stimulation (Fig. 6C). To ascertain whether cyclomulberrin's suppression of platelets was pharmacological or due to cytotoxic effects, we conducted the LDH cytotoxicity. As Fig. 6E indicated, cyclomulberrin remained non-toxic to platelets even at concentrations as high as 80 µM.

#### 3.4.2. Cyclomulberrin selectively inhibits ATP release and αIIbβ3 activation

**Fig. 7:**
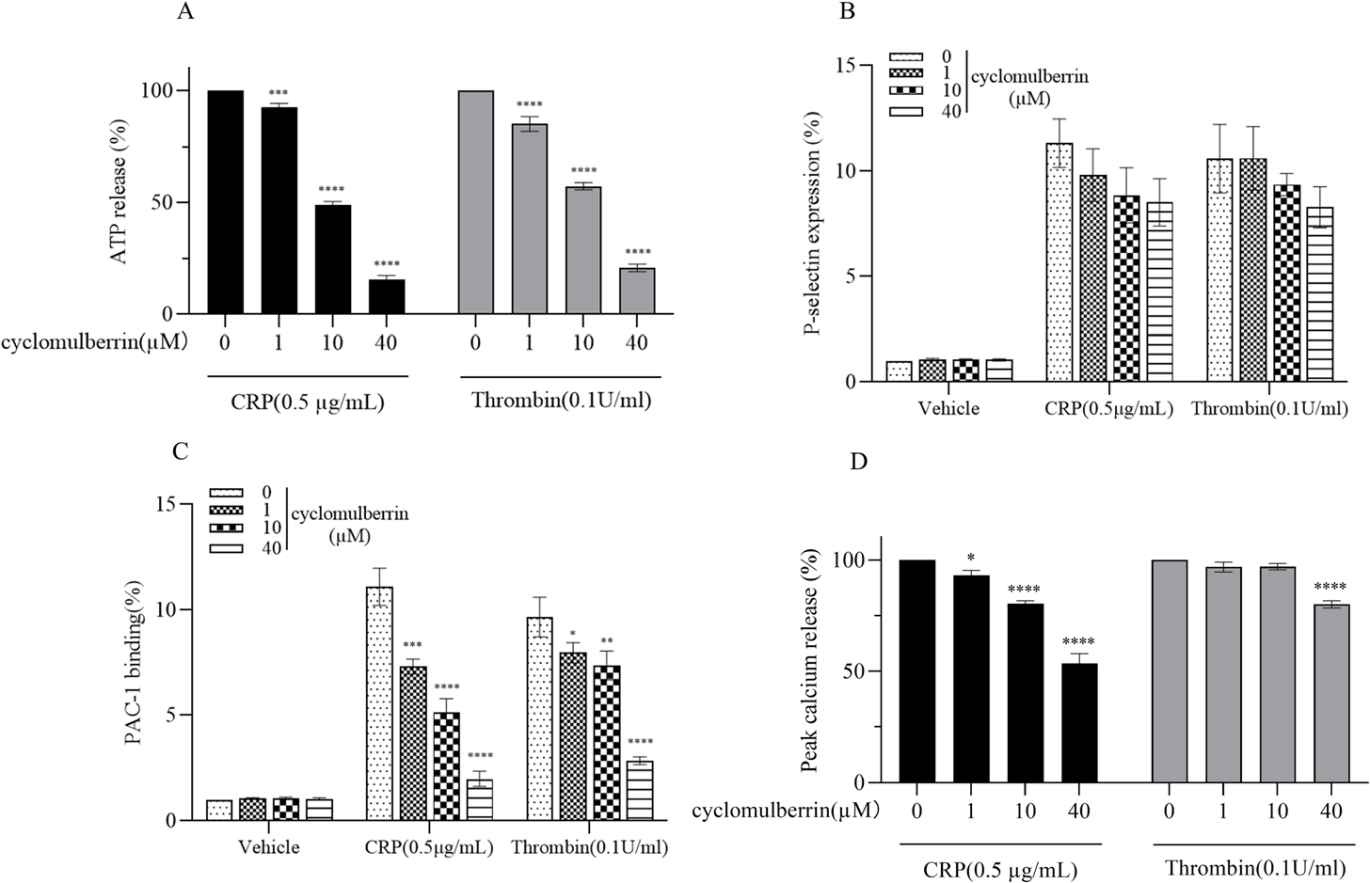
(A) Effect of cyclomulberrin on ATP release. (B) Effect of cyclomulberrin on P-selectin expression. (C) Effect of cyclomulberrin on αIIbβ3 activation. (D) Effect of cyclomulberrin on intracellular Calcium mobilization.

The granule content secretion in platelets and integrin αIIbβ3 activation are key processes in driving the positive feedback cycle of platelet activation.^41^ To explore cyclomulberrin's role in these processes, we assessed its impact on ATP release, P-selectin expression and αIIbβ3 activation. As depicted in Fig. 7A, cyclomulberrin displayed significant and concentration-dependent inhibitory effects on ATP release induced by both CRP and thrombin. In contrast, no significant changes in P-selectin expression were observed after cyclomulberrin treatment during CRP- or thrombin-stimulated platelet activation (Fig. 7B). Consistent with reduced platelet aggregation, cyclomulberrin dramatically reduced integrin αIIbβ3 activation as demonstrated by the decrease in PAC-1 binding to platelets (Fig. 7C). These findings suggested that cyclomulberrin inhibited platelet activation *via* selective effects on dense granule secretion and αIIbβ3 integrin activation, without impinging on platelet α-granule secretion.

#### 3.4.3. Cyclomulberrin modulates calcium mobilization in platelets

It is well known that Ca^2+^ mobilization in the cytosol is critical for platelet activation, which involves granule secretion and αIIbβ3 integrin activation.^42^ Hence, we explored the impact of cyclomulberrin on the mobilization of intracellular calcium levels. Fig. 7D indicated that during CRP-induced platelet activation, cyclomulberrin led to a significant and dose-dependent reduction in calcium levels. However, under thrombin-stimulated platelet activation, the calcium levels were only affected to a smaller degree (about 20%) after cyclomulberrin treatment at 40 µM (Fig. 7D).

#### 3.4.4. Cyclomulberrin suppresses ROS generation and increases cAMP levels in platelets

**Fig. 8:**
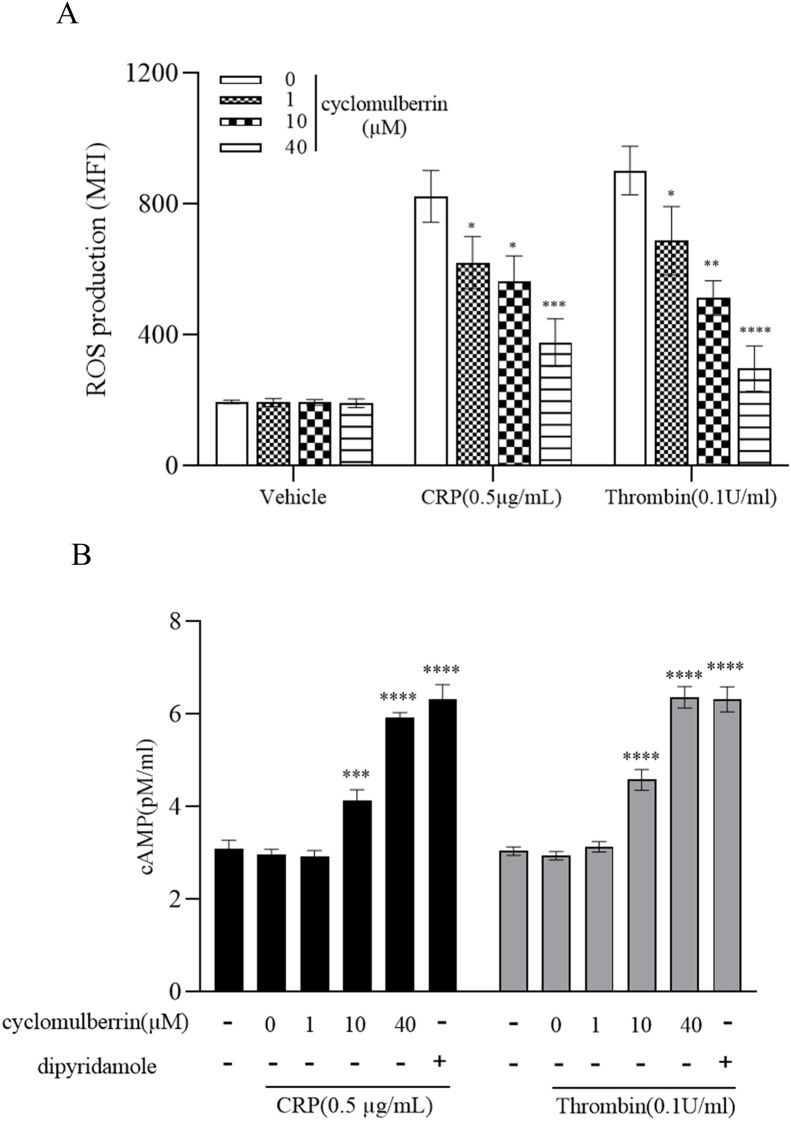
(A) Effect of cyclomulberrin on ROS generation. (B) Effect of cyclomulberrin and dipyridamole on cAMP generation.

Our GO enrichment analysis suggested a potential role for cyclomulberrin in countering oxidative stress and hydrogen peroxide reactions in thrombi. This prompted an investigation into cyclomulberrin's effect on platelet ROS production. As presented in Fig. 8A, cyclomulberrin significantly reduced ROS generation from platelets after stimulation with CRP and thrombin, underscoring its ant-oxidative properties in platelets. Recognizing that upregulation of cAMP in the platelet cytosol hinders platelet activation,^43^ and given our KEGG analysis suggested a cAMP signaling pathway involved in cyclomulberrin's antithrombotic effect, we turned our attention to its influence on platelet cAMP levels. Fig. 7B demonstrated that pretreatment with cyclomulberrin at 10 µM and higher dramatically enhanced cAMP levels in platelets activated by both CRP and thrombin. In addition, dipyridamole (20 µM), a phosphodiesterase (PDE) inhibitor, also significantly elevated platelet cAMP levels (Fig. 8B).

#### 3.4.5. Selective inhibition of SRC and AKT1 attenuates the anti-aggregatory effect of cyclomulberrin

**Fig. 9:**
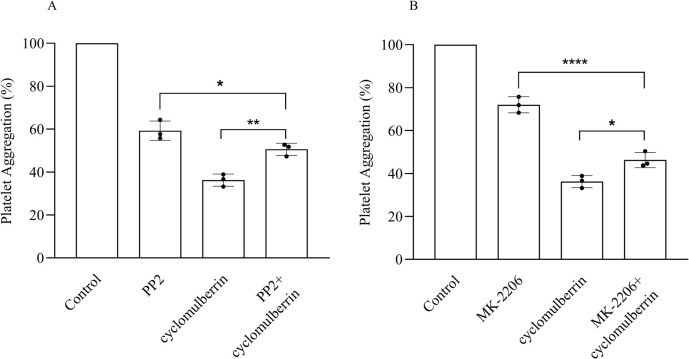
Functional target engagement validation: selective inhibitor attenuates cyclomulberrin's anti-aggregatory effect. (A) PP2 (SRC inhibitor). (B) MK-2206 (AKT inhibitor).

To test whether cyclomulberrin engages SRC and AKT1 – two hub targets in platelet activation – we used selective inhibitors PP2 (10 µM, SRC inhibitor) and MK-2206 (1 µM, AKT inhibitor) in CRP-stimulated human platelets. CRP-induced aggregation was set to 100% (control). Cyclomulberrin alone reduced aggregation to 36.24 ± 2.86%. PP2 alone gave 59.27 ± 4.48% aggregation – consistent with SFKs' role in GPVI-mediated platelet activation.^44,45^ Adding PP2 to cyclomulberrin raised aggregation to 50.60 ± 2.88%, significantly higher than cyclomulberrin alone (*P* < 0.01, Fig. 9A). Similarly, MK-2206 alone: 72.00 ± 3.71% aggregation; co-treatment with cyclomulberrin: 46.28 ± 3.54% (*P* < 0.05 *vs.* cyclomulberrin alone, Fig. 9B). Thus, cyclomulberrin suppresses platelet aggregation at least partly by engaging SRC and AKT1.

#### 3.4.6. Cyclomulberrin's influence on blood coagulation

Since the important regulatory role of platelets in blood coagulation^46^ and the association of cyclomulberrin with fibrinolysis suggested by our GO enrichment analysis, we undertook Prothrombin time (PT) and activated partial thromboplastin time (aPTT) assays to explore the influence of cyclomulberrin on blood coagulation. Based on Table 4, at concentrations below 40 µM, cyclomulberrin had no noticeable effect on PT and aPTT in comparison to the platelet poor plasma (PPP) group. However, at a concentration of 80 µM, cyclomulberrin significantly prolonged both PT and aPTT. These findings suggested that when used as an anti-thrombotic agent, cyclomulberrin exhibited a limited interference with the coagulation cascade, implying a reduced risk of bleeding side effects.

**Table 4:** Effect of cyclomulberrin on blood coagulation^a^

Samples	PT (sec)	aPTT (sec)
PPP	13.2 ± 0.1	35.6 ± 0.2
PPP + cyclomulberrin (1 µM)	13.0 ± 0.1 ^NS^	35.7 ± 0.2 ^NS^
PPP + cyclomulberrin (10 µM)	13.3 ± 0.1 ^NS^	35.8 ± 0.3 ^NS^
PPP + cyclomulberrin (40 µM)	13.4 ± 0.1 ^NS^	36.2 ± 0.3 ^NS^
PPP + cyclomulberrin (80 µM)	18.9 ± 0.1 ^****^	46.3 ± 0.3 ^****^

a The results were expressed as the mean ± standard deviation (*n* = 3). ^NS^, not significant *versus* PPP; ^****,^*P* < 0.0001 *versus* PPP.

## 4. Discussion

Cyclomulberrin, a flavonoid from *Morus alba*,^47,48^ has no previously reported antithrombotic activity. Using network pharmacology, we identified 241 compound-related targets and 1060 thrombus-associated genes, yielding 57 overlapping nodes and seven hub targets (TNF, AKT1, SRC, PTGS2, MMP9, PPARG, and CYCS). Their biological roles align with thrombosis: TNF and PTGS2 link inflammation to thrombosis: TNF induces vascular injury, whereas PTGS2 activates endothelium;^49,50^ SRC family kinases mediate rapid platelet response;^44^ AKT1 regulates matrix recognition and integrin activation;^51^ PPARG modulates plaque dynamics;^52,53^ MMP9 influences platelet protein degradation.^54^ KEGG enrichment pointed to four convergent pathways – platelet activation, PI3K-Akt signaling, cAMP signaling, and TNF cascade. These predictions suggest that cyclomulberrin modulates platelet activation *via* multi-target, multi-pathway mechanisms rather than *via* mono-pharmacological inhibition, awaiting experimental validation.

To directly test this hypothesis and provide credible target engagement evidence, we designed a two-tier validation strategy. Tier 1 tested whether cyclomulberrin engages representative hub targets functionally. Selecting SRC and AKT1 for their established centrality in platelet signaling, we applied selective inhibitors PP2 and MK-2206 in CRP-stimulated platelets. Both compounds attenuated cyclomulberrin's anti-aggregatory effect—PP2 profoundly, MK-2206 partially—demonstrating that these kinases are indeed engaged in the drug's mechanism (Section 3.4.5, Fig. 9). The incomplete rescue implies simultaneous action through additional hubs (TNF, PTGS2, PPARG), consistent with the network prediction of multi-target engagement. Tier 2 examined binding plausibility for all seven hub targets. We performed docking with site-specific grids centered on orthosteric pockets, achieved redocking validation (RMSD < 2.0 Å), and extended each complex to 100-ns MD simulations. Converged RMSD trajectories, stable SASA values, favorable MM/PBSA energies, and persistent hydrogen bonds across all seven systems support the capacity for physical engagement (Section 3.3.2, Table 3). Compared to native ligands (SI Fig. S8), cyclomulberrin occupies the same pockets but forms distinct interaction networks, with partial residue overlap for MMP9, PPARG, and SRC –suggesting distinct binding modes of target engagement.

With target engagement supported, we examined functional consequences in platelets. Cyclomulberrin notably decreased CRP- and thrombin-induced aggregation, guiding our choice of agonists for subsequent tests. The compound selectively inhibited ATP release and αIIbβ3 activation without affecting α-granule secretion, indicating targeted modulation of dense granule exocytosis and integrin signaling, rather than non-specific global secretory suppression. Intracellular Ca^2+^ mobilisation dropped sharply (especially under CRP stimulation), while cAMP levels increased – an increase indicating engagement of the protective cAMP signaling pathway.^43^ ROS scavenging activity accompanied these effects. Notably, coagulation parameters remained normal below 40 µM. distinguishing cyclomulberrin from agents that compromise hemostasis. Collectively, these cellular phenotypes corroborate Tier 1 findings of SRC and AKT1 engagement; however, the incomplete rescue profile mandates parallel contributions from other hubs (*e.g.*, TNF, PPARG).^50,53^

**Fig. 10:**
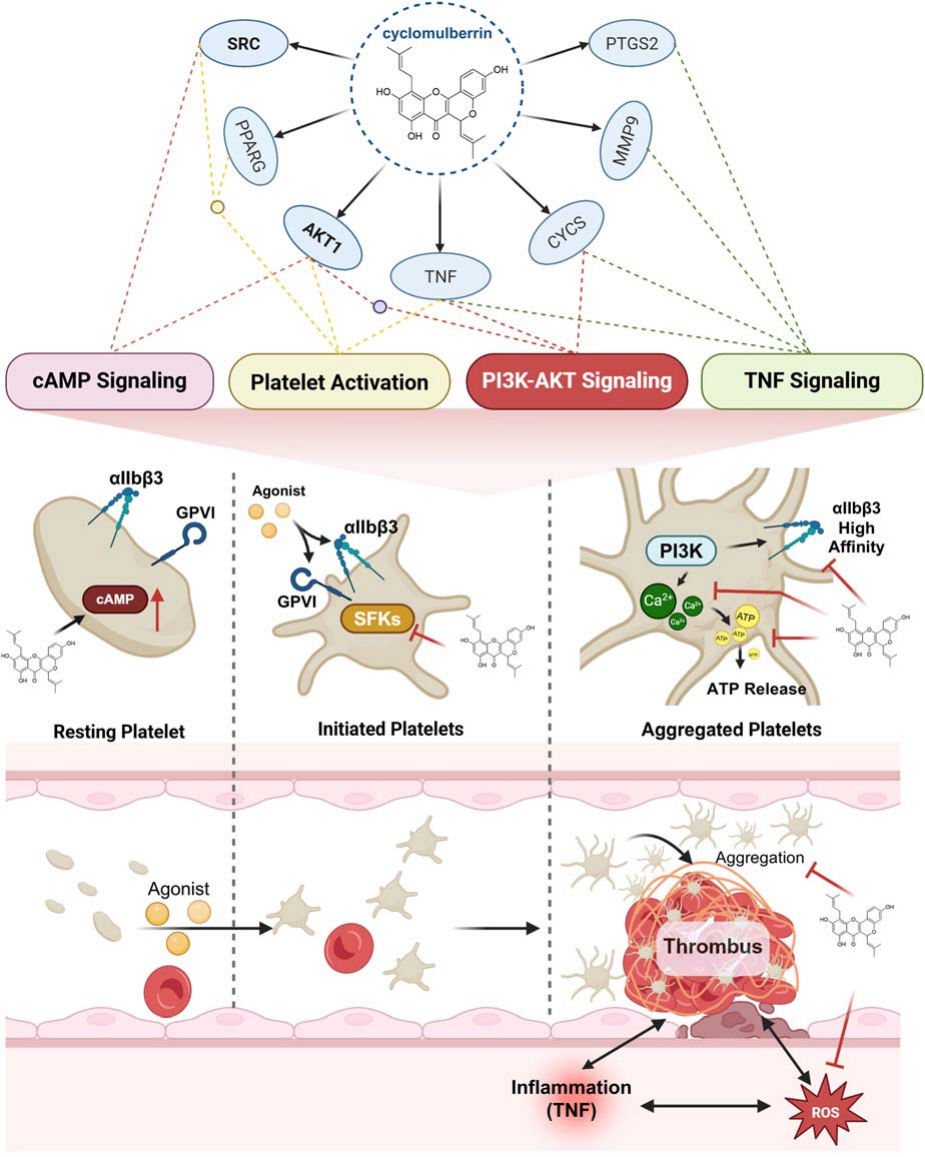
Proposed mechanism of cyclomulberrin in inhibiting platelet activation *via* multi-target and multi-pathway regulation.

Fig. 10 integrates these findings into a coherent mechanistic model. Cyclomulberrin engages seven hub targets, converging on four signaling axes that govern platelet activation – from resting state through initiation to aggregation.^55,56^ At the cellular level, this multi-target engagement suppresses Ca^2+^ flux, ATP release, and αIIbβ3 activation while elevating cAMP. At the vascular level, ROS scavenging disrupts the oxidative stress-thrombosis-inflammation network^57–60^ at injured endothelium. This architecture distinguishes cyclomulberrin from single-target antithrombotics and explains why SRC or AKT1 inhibition alone cannot fully restore aggregation.

It should be noted that this study has three limitations. First, network pharmacology predictions may include false positives or overlook key targets.^61^ Second, although functional and computational evidence supports engagement of all seven hubs, direct biophysical confirmation (SPR, CETSA)^62,63^ is still needed. Third, all data are *in vitro*, and the findings require validation in *in vivo* models. Despite these limitations, convergent *in silico* and *in vitro* evidence conclusively demonstrates that cyclomulberrin modulates platelet activation *via* multi-target, multi-pathway mechanisms, validating the network pharmacology prediction.

## 5. Conclusions

Integrating network pharmacology, computational simulations, and functional assays, we demonstrate that cyclomulberrin, a flavonoid isolated from *Morus alba*, exerts antithrombotic activity through a multi-target, multi-pathway mechanism. Network pharmacology identified seven hub targets (TNF, AKT1, SRC, PTGS2, MMP9, PPARG and CYCS) enriched in platelet activation, PI3K-Akt, cAMP, and TNF pathways. Molecular docking and 100-ns MD simulations confirmed stable binding of cyclomulberrin to all seven targets, and selective inhibitor experiments validated functional engagement of SRC and AKT1. Cyclomulberrin inhibited platelet aggregation, αIIbβ3 activation, ATP release, Ca^2+^ mobilisation and ROS generation, while elevating cAMP levels, without affecting coagulation at concentrations up to 40 µM. These findings establish cyclomulberrin as a plant-derived, multi-target antiplatelet agent. As the most logical next step, validation in a murine model of arterial thrombosis (FeCl_3_-induced carotid artery injury) will be essential to confirm antithrombotic efficacy and to assess key mechanistic targets (*e.g.*, SRC, AKT1) and coagulation parameters *in vivo*, thereby further supporting its development as an antithrombotic agent.

## Institutional review board statement

The study was conducted in accordance with the Declaration of Helsinki, and approved by the Medical Ethics Committee of Yuebei People's Hospital (KY-2021-221, 2021).

## Informed consent statement

Informed consent was obtained from all subjects involved in the study.

## Author contributions

Conceptualization, Q. L. and Y. G.; methodology, Q. L. and J. P.; investigation, Y. G.; formal analysis, Y. G., S. Y. and R. H.; writing – original draft preparation, Q. L. and Y. G.; writing – review & editing, R. H., Y. M. and J. P. All authors have read and agreed to the published version of the manuscript.

## Conflicts of interest

There are no conflicts to declare.

## Supplementary Material

RA-OLF-D6RA00546B-s001

## Data Availability

The data supporting this article have been included as part of the supplementary information (SI). Supplementary information: S1: procedure of extraction and isolation; S2–S3: ^1^H and ^13^C NMR spectra of cyclomulberrin (in actone-d_6_); S4–S7: ethical approval document and informed consent form (each in Chinese with English translation); S8–S11: site-specific docking results (binding pose comparisons, RMSF, FEL, energy analysis); S12–S19: original blind docking results (protocol, affinities, poses, MD analyses). See DOI: https://doi.org/10.1039/d6ra00546b.
