# Supplementary material for: Cyclomulberrin from Morus alba L. exerts antithrombotic effects by modulating platelet activation: an integrated network pharmacology and in vitro study
Source: RSC Adv. 2026 Jul 2;16(34):32373–84. doi: 10.1039/d6ra00546b (PMC13325654; doi:10.1039/d6ra00546b)
Supplement: RA-016-D6RA00546B-s001 [file RA-016-D6RA00546B-s001.pdf]

Supplementary Information (SI) for RSC Advances.
This journal is © The Royal Society of Chemistry 2026

Supplementary Information

Cyclomulberrin from *Morus alba* L. exerts antithrombotic effects by modulating platelet activation: an integrated network pharmacology and in vitro study

Yanqiong Guo* [a], Rongrong He [a], Jing Peng [a], Shuguo Yuan [b], Yanlei Ma [a], and Qingde Li [a]

[a] *Department of Pharmacy, Yuebei People's Hospital, Shantou University Medical College, Shaoguan, 512026, China;*
[b] *Department of Cardiology, Yuebei People's Hospital, Shantou University Medical College, Shaoguan, 512026, China;*
Corresponding author: Qingde Li
E-mail: gyqiong@mail2.sysu.edu.cn

S1. Procedure of extraction and isolation.

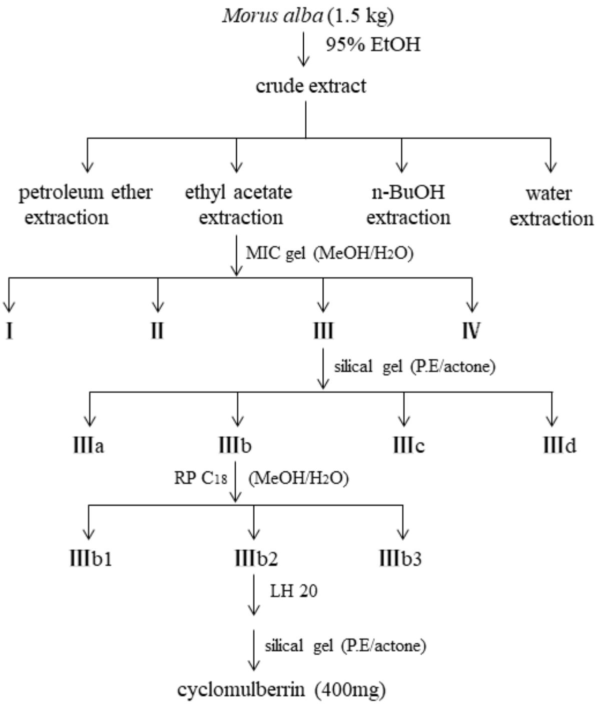

S2.^{1}H NMR (400 MHz, Acetone-d_6) spectrum of cyclomulberrin

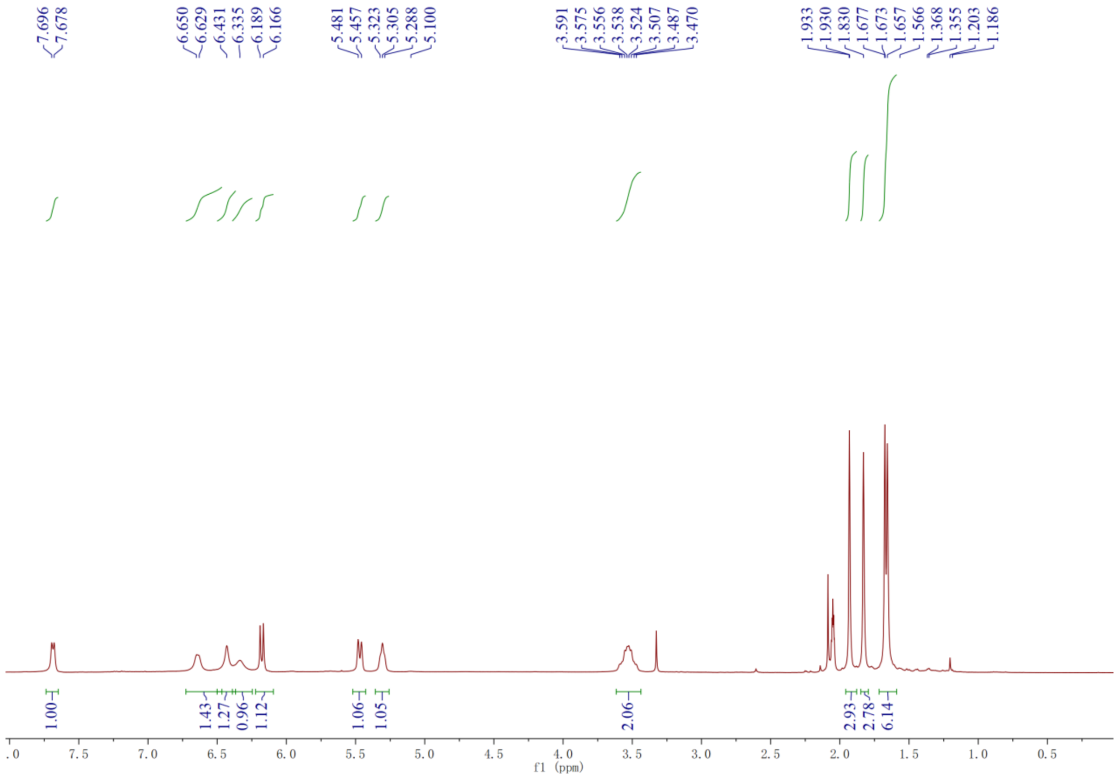

S3. ^{13}C NMR (100 MHz, actone-d_6) spectrum of cyclomulberrin

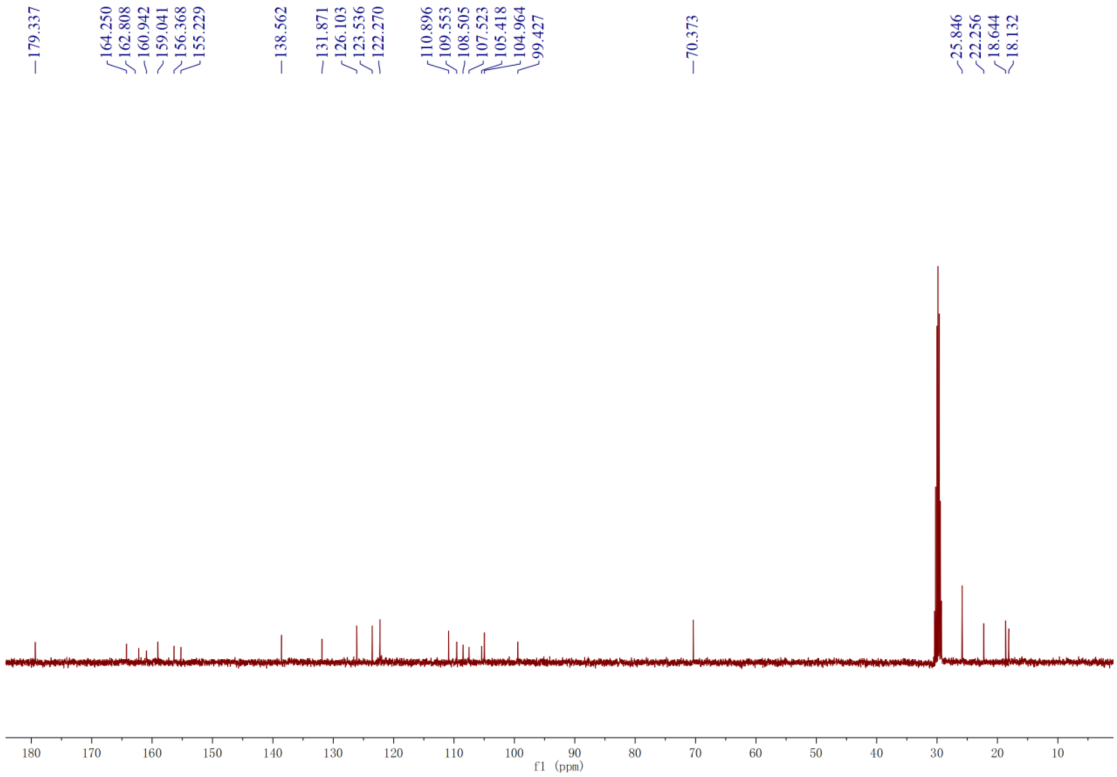

S4. Ethical Approval Document in Chinese

粤北人民医院伦理审查表

申请日期：2021 年 8 月 31 日　　　　**所在单位：**粤北人民医院

项目名称：　桑白皮黄酮抗血栓作用及其机制研究

项目负责人：郭艳琼　联系电话：15902029775　邮箱：1455821039@qq.com

合作研究单位：　　　　负责人：　　　　联系电话：

研究组主要成员：郭艳琼；何蓉蓉；彭晶；李庆德

请求审查类型：☑申请项目　☐批准后项目　☐延续项目　☐委托项目　☐其他

研究项目来源：

☐纵向课题（☐国家自然科学基金、☐广东省省级财政科研项目等）

☐横向课题　☐研究生课题　☑自选课题　☐其他

研究起止时间：2022 年 1 月 1 日-2023 年 12 月 31 日

递交审查资料

☐研究方案　☐知情同意书　☐调查问卷　☐其他资料　☐论文

包括：试验用品安全性资料、生产企业资质证明、试验用品提供者的资质证明。

涉及人的生物医学研究内容及研究方案摘要（200 字以内）

目的：探讨桑白皮黄酮对血小板功能及抗血栓机制。**方法：**筛选 18-60 岁健康志愿者 4-10 名，满足特定健康标准，采集每人 2-5ml 静脉血。经梯度离心法提取血小板后展开血栓实验。**主要指标：**桑白皮黄酮对血小板聚集、颗粒物分泌、钙流动性、αIIbβ3 激活、ROS 和 cAMP 生成、凝血功能等的作用。**预期：**揭示桑白皮黄酮的抗血栓机制，为抗血栓药物研发提供依据。以上所有实验在严格条件下进行，确保数据可靠与准确。

项目负责人签字：郭艳琼 郭艳琼　　　　**2021 年 8 月 31 日**

批件号：KY-2021-221

伦理委员会意见

处理意见：☐会议审查　☑快速审查

审查意见：☑通过　☐不通过

主任委员签字：[seal]　　　　（盖章）

2021 年 10 月 8 日

S5. Ethical Approval Document (English Translation)

Yuebei People's Hospital Medical Ethics Review Form

Application Date: August 31, 2021 **Research Unit:** Yuebei People's Hospital

**Project Name:** Study on the Anti-thrombotic Effect and Mechanism of Flavonoids form *Morus alba*
**Project Leader:** Yanqiong Guo **Contact Number:** 15902029775<br>**Email:**1455821039@qq.com
**Collaborative Research Unit:** **Leader:** **Contact Number:**
**Team Members:** Yanqiong Guo; Rongrong He; Jing Peng; Qingde Li
**Type of Review Requested:**<br>☑New Project ☐ Ongoing Project ☐ Extended Project<br>☐Entrusted Project ☐Other (please specify)
**Source of Research Project:**<br>☐Vertical Project (National Natural Science Foundation of China, Guangdong Provincial Government Research Fund, etc)<br>☐Horizontal Project ☐Graduate Project ☑Self-selected Project ☐Other
**Research Duration:** January 2022 - December 2023
**Submitted Review Materials:**<br>☑Research Plan ☑ Informed Consent Form ☐ Survey Questionnaire<br>☐Thesis ☐Other (please specify)<br>Including: Safety data of test products, qualification certificate of the manufacturer, qualification certificate of the test product provider.

**Summary of Biomedical Research Involving Humans (within 200 words):**<br>**Objective:** Explore the anti-thrombotic effect and mechanism of flavonoids form *Morus alba*. **Method:** Select 4-10 healthy volunteers aged 18-60, meeting specific health criteria, and collect 2-6ml of venous blood from each. After extracting platelets using gradient centrifugation, thrombosis experiments are conducted. **Main Indicators:** Effects of flavonoids on platelet aggregation, granule secretion, calcium mobilization, αIIbβ3 activation, ROS and cAMP generation, and coagulation function, etc. **Expected Outcome:** Reveal the anti-thrombotic mechanism of flavonoids form *Morus alba*, providing a basis for the development of new anti-thrombotic drugs. All experiments are conducted under strict conditions to ensure data reliability and accuracy.
**Signature of Applicant:** **Date:2021-8-31**
**Ethics Committee Opinion**<br>**Processing Opinion**： ☐Full Committee Review ☐Expedited Review<br>**Review Opinion**： ☐Approved ☐Not Approved<br><br>**Signature of Chairperson**： **Date:**

S6. Informed Consent Form in Chinese

知情同意书

受试者须知

尊敬的________：

您被邀请参与由主要研究者郭艳琼负责的"桑白皮黄酮抗血栓作用及其机制研究"。此研究由粤北人民医院支持，资金来源于韶关市卫生健康科研项目(Y22096)和广东省中医药局科研项目 20232174)。请您仔细阅读以下内容，如有疑问请提出。

您参加本项研究是自愿的。本次研究已通过粤北人民医院医学伦理委员会审查。

研究目的：研究桑白皮里的黄酮类化合物对血栓的抑制作用和机制。

研究过程：参与者将被编号并建立研究档案。专业人员将从您的胳膊上抽取 2.0~5.0 毫升静脉血，共 1 次。从此血样本提取血小板后仅用于本项目后续抗血栓实验研究。

风险与不适：采集过程将严格按照无菌要求操作，可能导致短暂疼痛或局部青紫。极少数情况下可能出现针头感染。我们已制定了详细的风险预案应对这些潜在风险。

受益：您将获得项目补助 100 元，您的参与将为新型抗血栓药物的研发提供重要数据。

受试者职责：提供有关自身病史和当前身体状况的真实情况，及时报告任何不适，告知是否正参与其他研究。

隐私保护：您的个人信息和样本将被严格保密。样本将以编号标识，不含姓名。只有研究团队可访问您资料。研究结果公布时，不会透露任何个人信息。

您有权随时退出研究，不会受到任何影响。如有疑问，可联系郭艳琼（15902029775）或粤北人民医院医学伦理委员会（0751-6913515）。

知情同意签字页

我已了解研究的所有信息，并有机会提问。我自愿参与此研究，并知道我可以随时退出。我将收到此一份包含我和研究者的签名的"知情同意书"副本。

受试者姓名：__________　　联系电话：__________

受试者签名：__________

日期：______年______月______日

我已准确地将这份文件告知受试者，他/她准确地阅读了这份知情同意书，并有机会提出问题。

研究者姓名：__________　　联系电话：__________

研究者签名：__________

日期：______年______月______日

（注：如受试者不识字，需见证人签名；如受试者无行为能力，需代理人签名）

S7. Informed Consent Form (English Translation)

Informed Consent Form

Participant Information Sheet

Dear Participant________

You are invited to participate in the research titled "Study on the Anti-thrombotic Effect and Mechanism of Flavonoids form *Morus alba*" , led by Principal Investigator Yanqiong Guo. This research is supported by Yuebei People's Hospital and funded by the Shaoguan Health Bureau Research Fund (No. Y22096) and the Administration of Traditional Chinese Medicine of Guangdong Province (NO. 20232174). Please read the following information carefully and ask questions if any arise.

Your participation in this study is voluntary. This research has been reviewed and approved by the Medical Ethics Committee of Yuebei People's Hospital.

Research Objective: To study the inhibitory effect and mechanism of flavonoids extracted from *Morus alba* L. on thrombosis.

Research Process: Participants will be assigned a unique identification number and a study file will be established. A trained professional will collect 2.0 - 5.0 ml of venous blood from your arm once. Platelets extracted from this blood sample will be used exclusively for subsequent antithrombotic experimental studies within this research project.

Risks and Discomfort: The collection procedure will adhere strictly to aseptic techniques. It may cause brief pain or local bruising at the needle insertion site. In extremely rare cases, needle-stick infection may occur. We have established detailed risk management protocols to address these potential risks.

Benefits: You will receive a participation stipend of 100 RMB. Your participation will provide important data for the research and development of new anti-thrombotic drugs.

Participant's Responsibilities: Provide truthful information about your medical history and current health status, promptly report any discomfort, and disclose participation in other studies.

Privacy Protection: Your personal information and biological samples will be strictly confidential. Samples will be labeled only with your study identification number, not with your name. Only the authorized research team will have access to your data. No personal information will be disclosed when study results are published.

You have the right to withdraw from the study at any time without repercussions. For inquiries, contact Yanqiong Guo (15902029775) or the Medical Ethics Committee of Yuebei People's Hospital (0751-6913515).

Informed Consent Signature Page

I have read all the information provided about this study and have had the opportunity to ask questions. I voluntarily agree to participate in this research and understand that I may withdraw at any time. I will receive a copy of this "Informed Consent Form" signed by both myself and the investigator.

Participant Name: __________ Contact Number: __________

Participant Signature: __________

Date: ______/______/______

I have accurately explained this document to the participant. He/She has accurately read this informed consent form and has had the opportunity to ask questions.

Researcher Name: __________ Contact Number: __________

Researcher Signature: __________

Date: ______/______/______

(Note: If the participant is illiterate, a witness signature is required. If the participant lacks the capacity to consent, a legal guardian/proxy signature is required.)

S8. Comparison of binding poses of cyclomulberrin (blue) with co-crystallized native ligands (brown) for each target (site-specific docking).

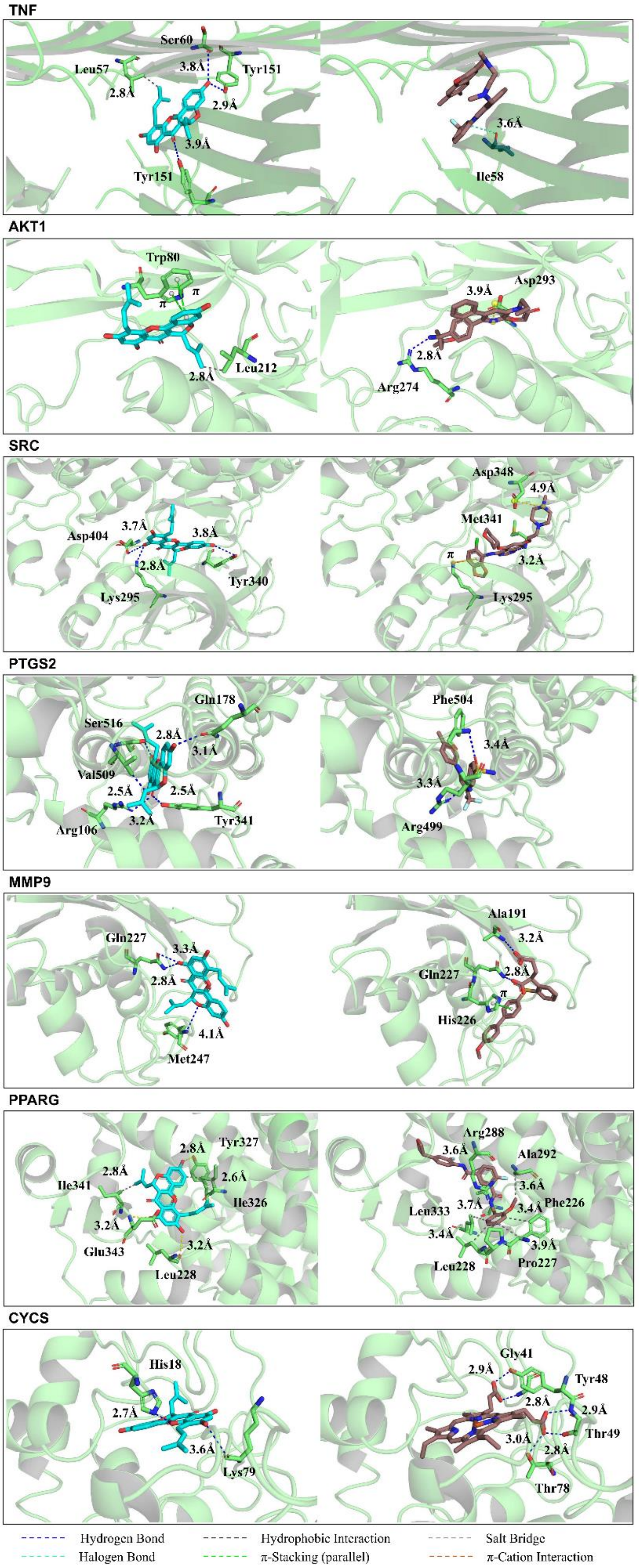

S9. Root-mean-square fluctuation (RMSF) for the seven cyclomulberrin-target complexes (site-specific docking).

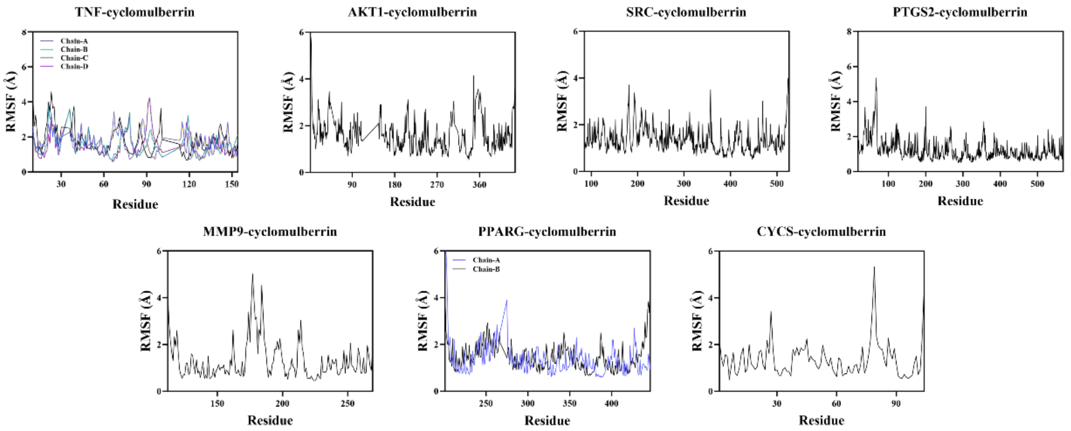

S10. Free energy landscapes (FEL) for the seven cyclomulberrin-target complexes (site-specific docking).

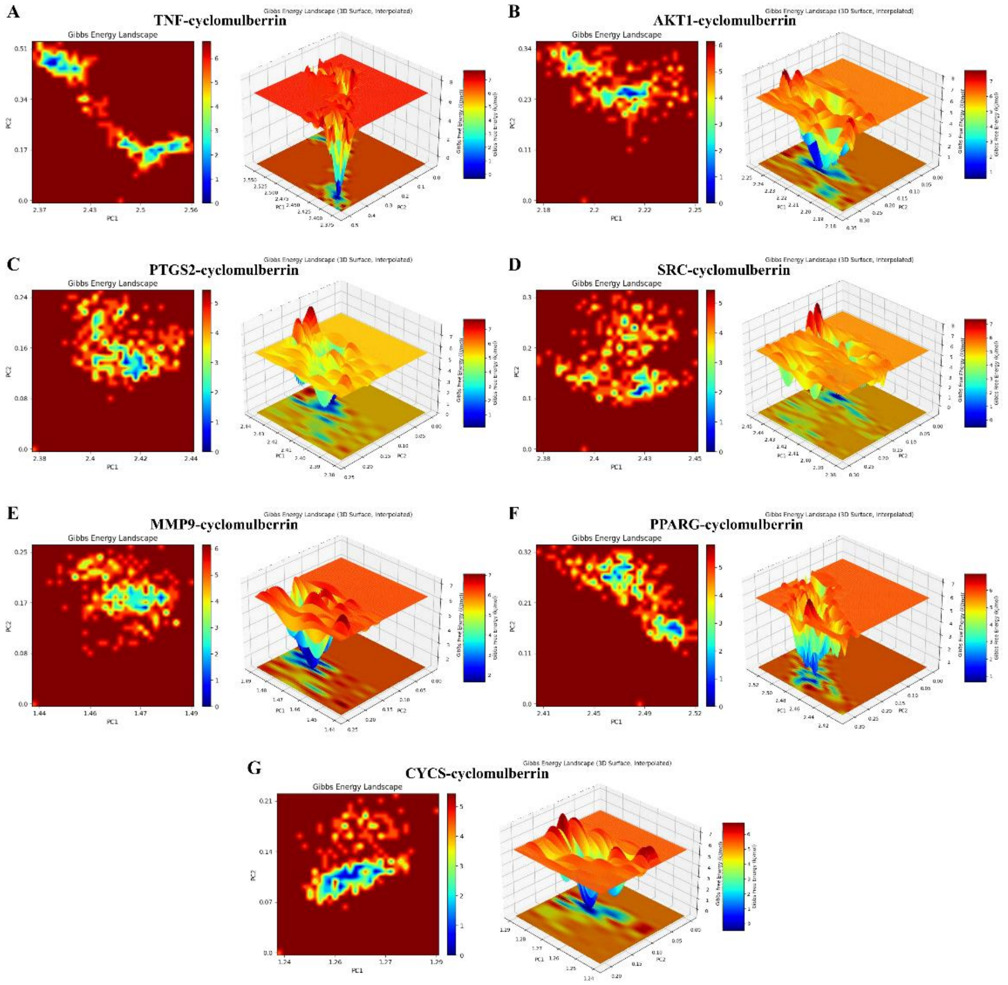

S11. Dynamic energy analysis for the seven cyclomulberrin-target complexes (site-specific docking): (A) Binding free energy components; (B–H) Per-residue energy decomposition for each complex.

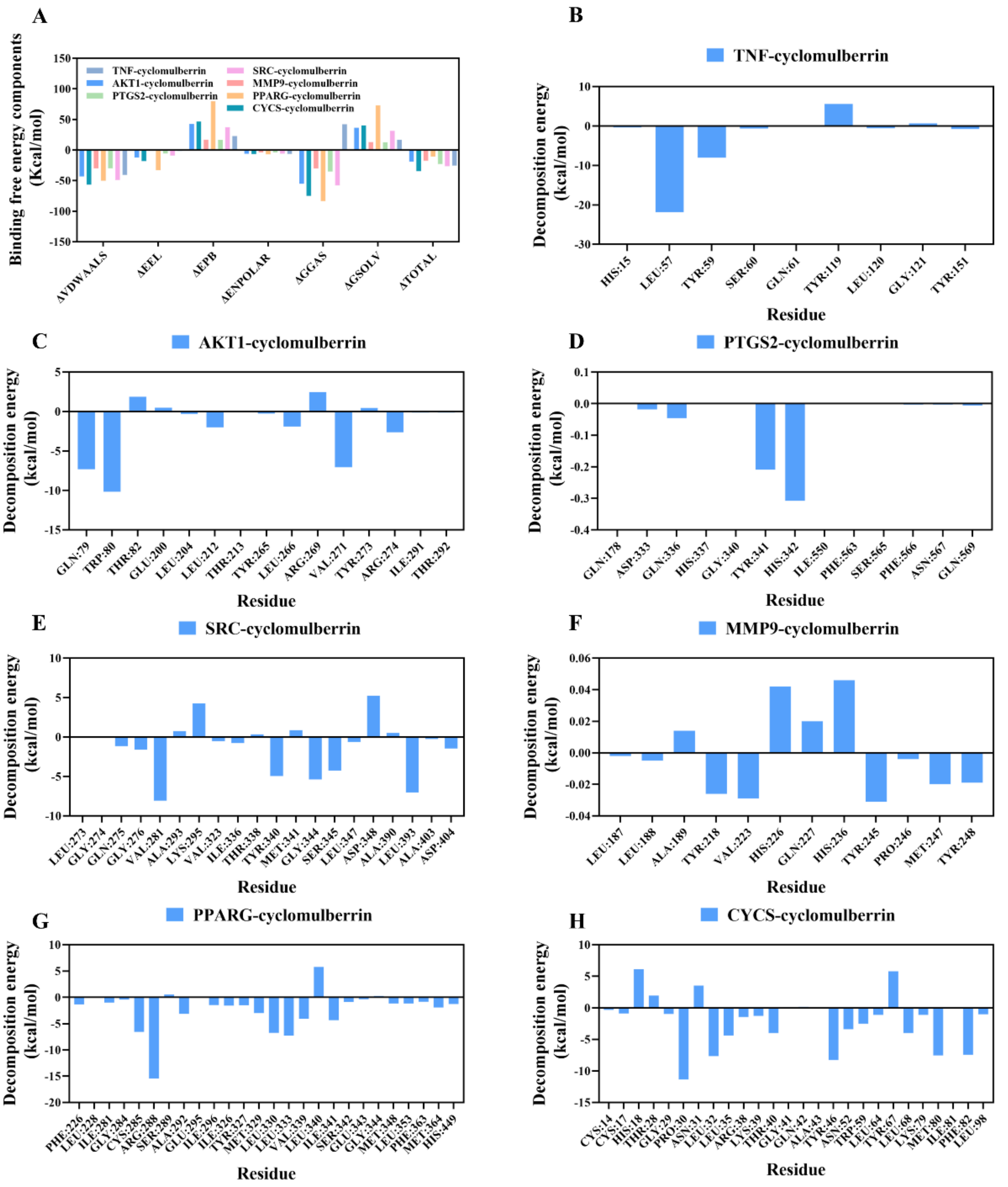

S12. Original blind docking (exploratory, not used for main conclusions): detailed protocol, binding affinities (kcal/mol), and binding poses of cyclomulberrin with seven hub targets.

Method. The interaction affinities and modes between cyclomulberrin and the identified hub targets were explored using AutodockVina 1.2.2 software (https://www.dockeasy.cn/,). Hub targets 3D coordinates were retrieved from the PDB database (http://www.rcsb.org/). For the docking, both protein and cyclomulberrin data were converted into PDBQT format after removing water molecules and adding polar hydrogen atoms. The grid box was centered on each protein's domain to accommodate free molecular movement. The docking pocket was set up as a 30 Å × 30 Å × 30 Å square spaces with a grid point distance of 0.05 nm. Finally, the results were analyzed and visualized using PyMol.

Binding affinities were shown in table below:

Target	PDB ID	Unitprot	Description	Ligand	Binding affinity/(kcal/mol)
TNF	2E7A	P01375	Tumor necrosis factor	cyclomulberrin	-9.49
AKT1	7NH5	P31749	RAC-alpha serine/threonine-protein kinase	cyclomulberrin	-7.64
SRC	7NG7	P12931	Proto-oncogene tyrosine-protein kinase Src	cyclomulberrin	-7.56
PTGS2	5F19	P35354	Prostaglandin G/H synthase 2	cyclomulberrin	-9.17
MMP9	6ESM	P14780	Matrix metalloproteinase-9	cyclomulberrin	-5.03
PPARG	8B92	P37231	Peroxisome proliferator-activated receptor gamma	cyclomulberrin	-9.66
CYCS	5TY3	P99999	Cytochrome c	cyclomulberrin	-6.38

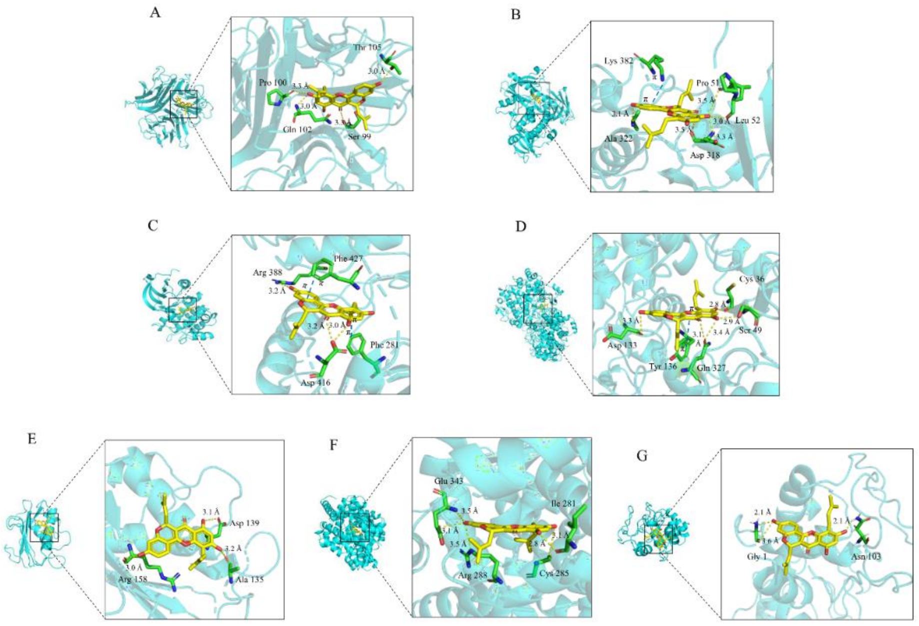

Fig. Binding modes and interactions of cyclomulberrin with the top seven hub targets. Binding mode and interactions of cyclomulberrin with TNF (A), AKT1 (B), SRC (C), PTGS2 (D), MMP9 (E), PPARG (F) and CYCS (G).

S13. Original blind docking: RMSD profiles of five representative cyclomulberrin-target complexes.

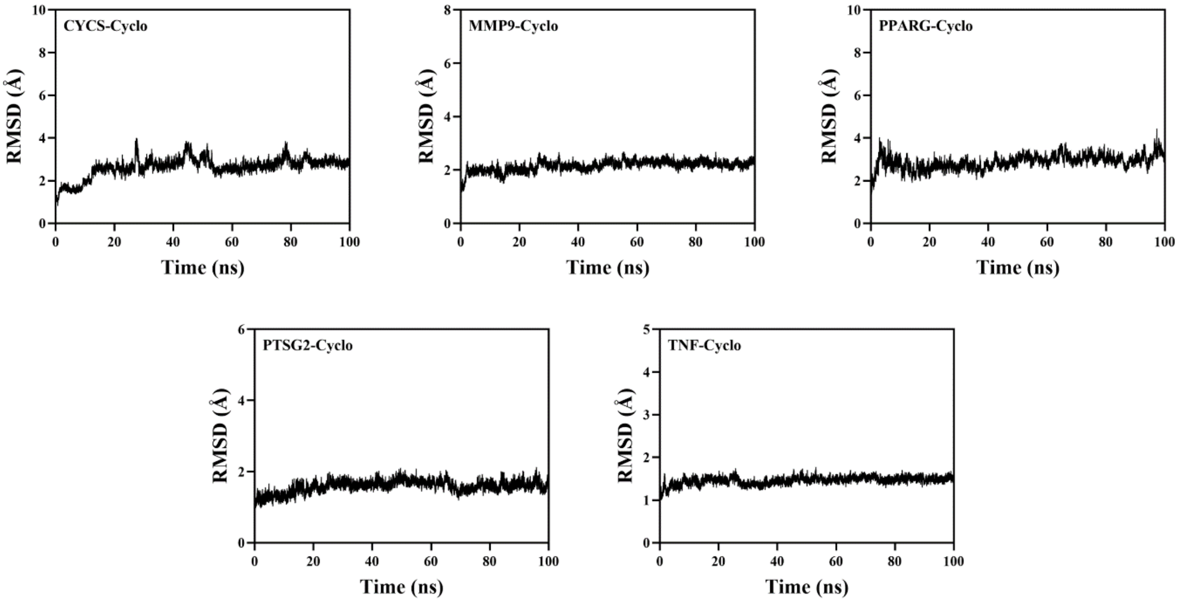

S14. Original blind docking: Rg for the five complexes.

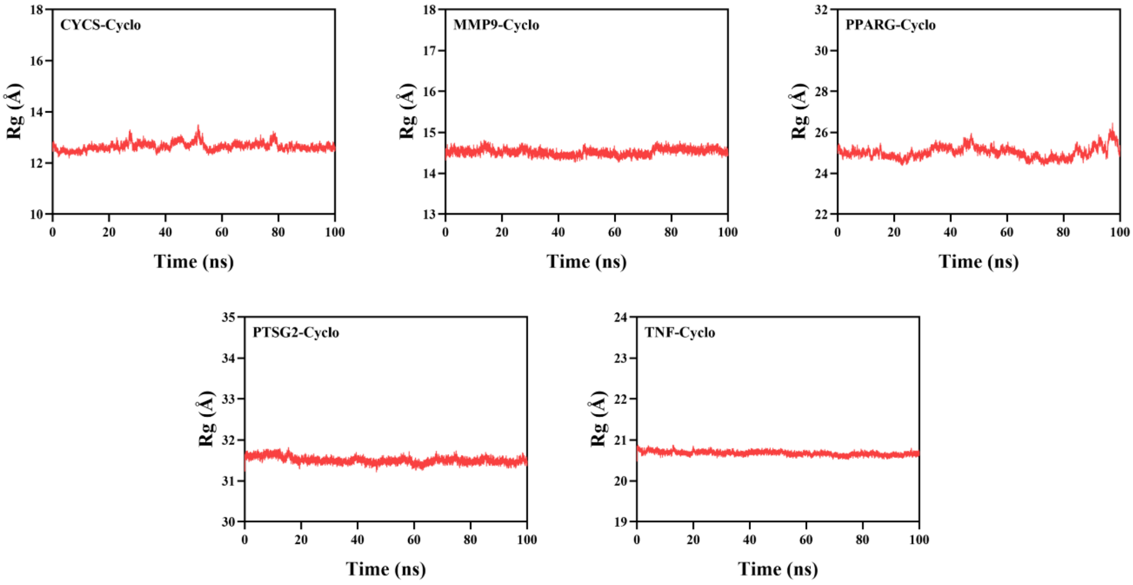

S15. Original blind docking: SASA for the five complexes.

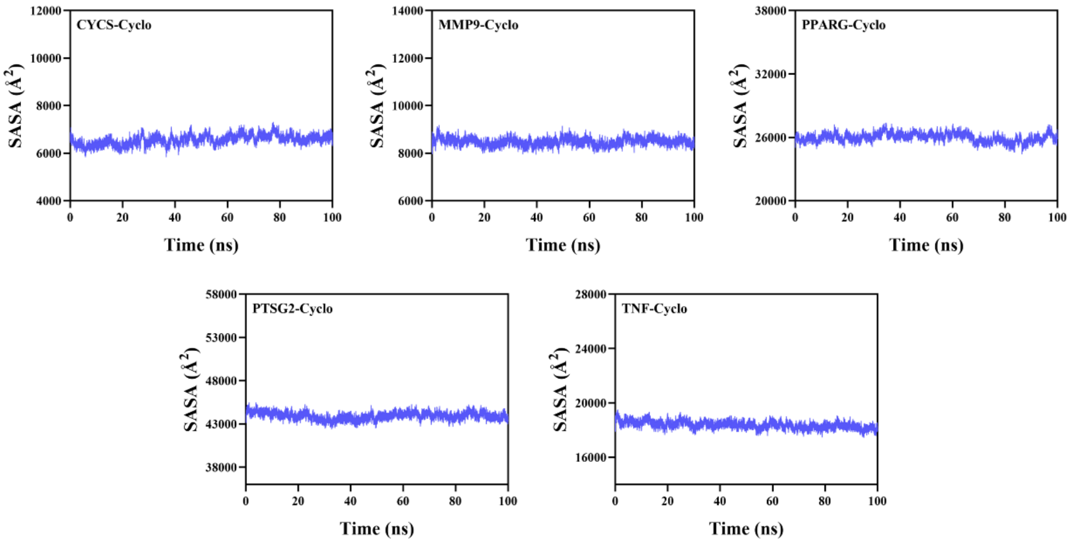

S16. Original blind docking: number of hydrogen bonds during MD simulations for the five complexes.

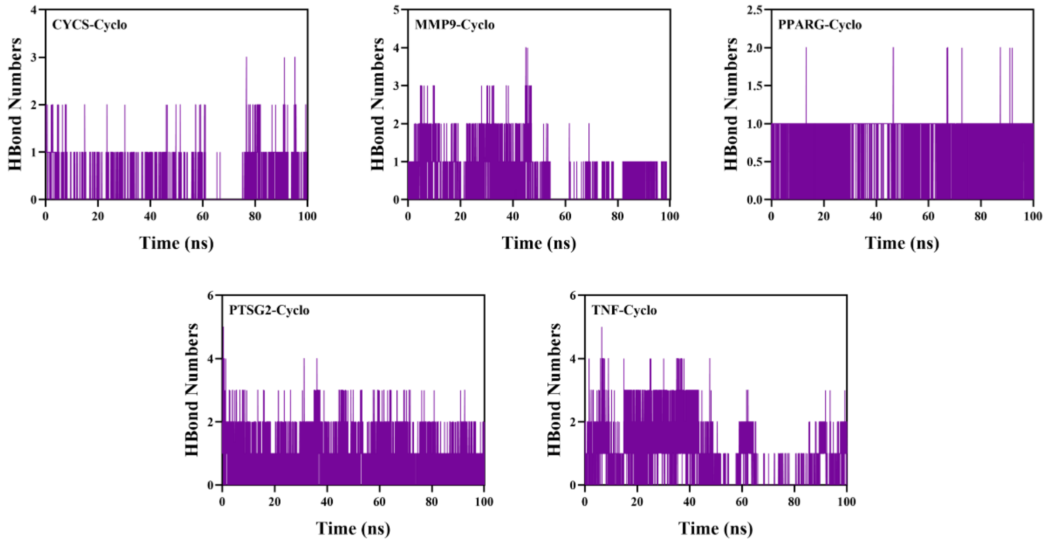

S17. Original blind docking: RMSF for the five complexes.

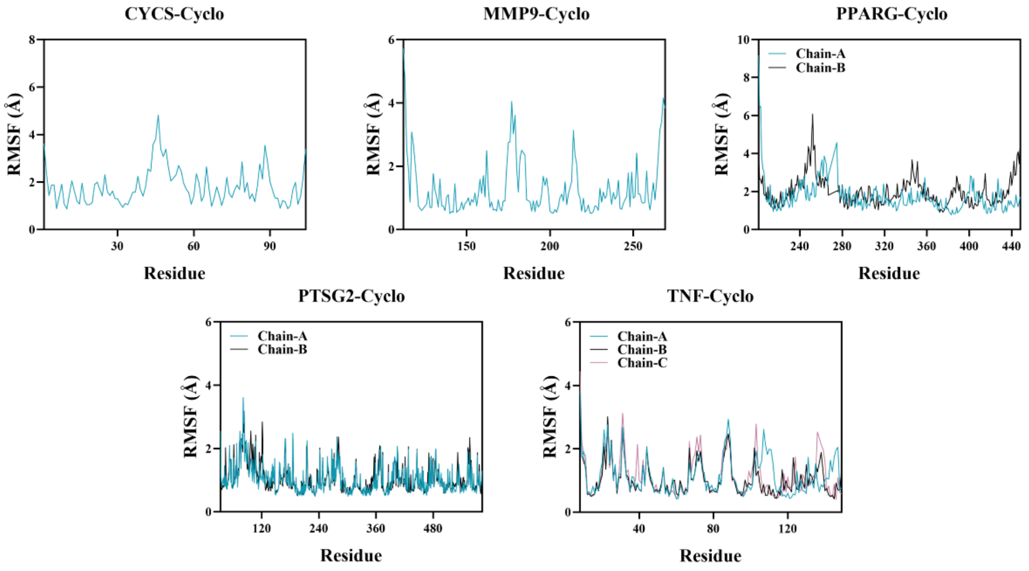

S18. Original blind docking: free energy landscapes for the five complexes.

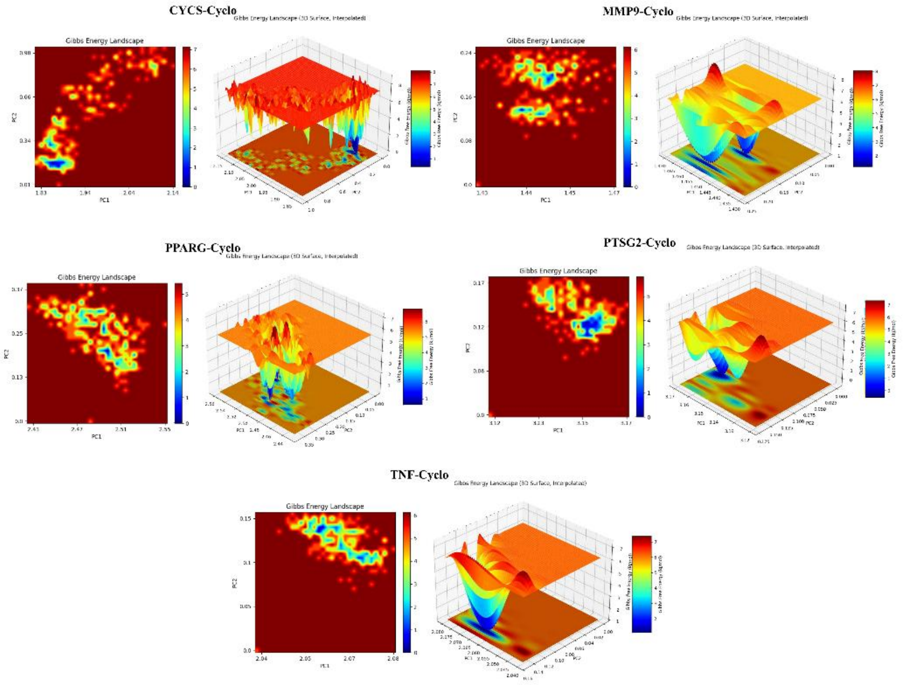

S19. Original blind docking: dynamic energy analysis of the five complexes (table and figure)

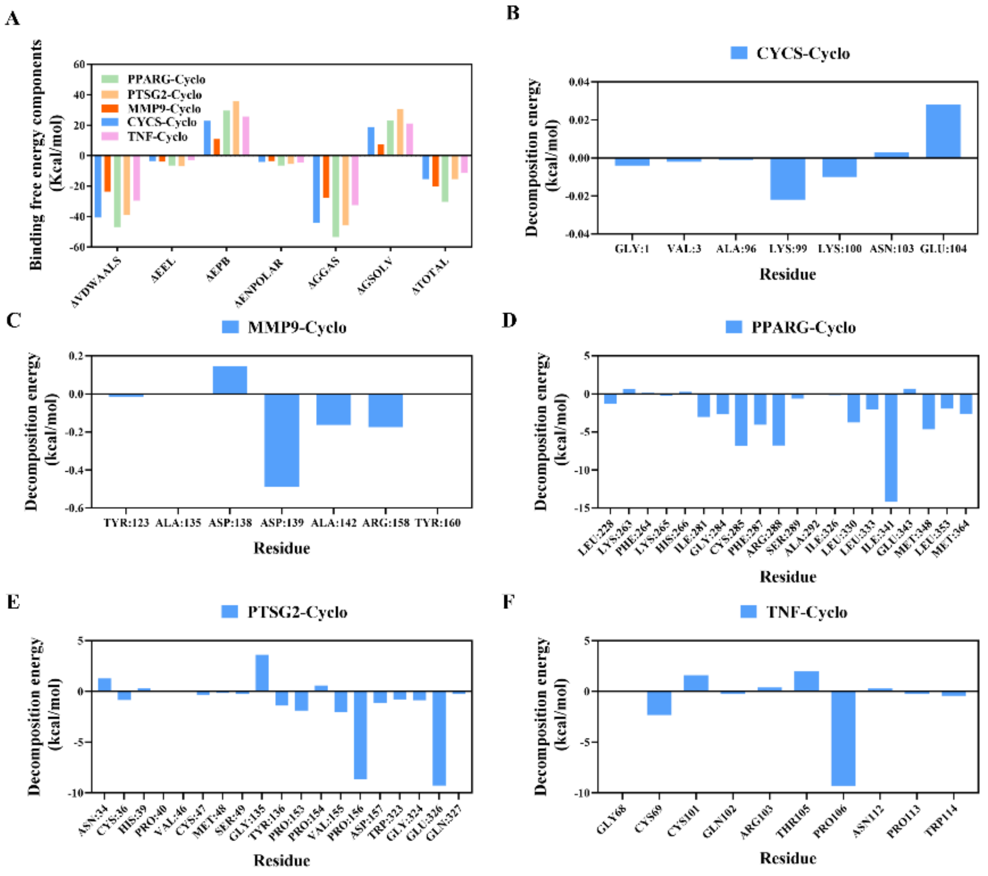

Table Dynamic energy analysis of the five complexes

Energy components	CYCS-Cyclo	MMP9-Cyclo	PPARG-Cyclo	PTSG2-Cyclo	TNF-Cyclo
ΔVDWAALS	-30.73 ± 3.88	-23.66 ± 1.66	-46.96 ± 3.26	-39.00 ± 3.94	-29.53 ± 4.91
ΔEEL	-3.53 ± 1.46	-3.96 ± 1.89	-6.50 ± 2.96	-6.85 ± 2.58	-3.00 ± 3.68
ΔEPB	22.93 ± 3.20	10.96 ± 2.06	29.60 ± 4.67	35.89 ± 4.43	25.71 ± 5.55
ΔENPOLAR	-4.14 ± 0.47	-3.51 ± 0.27	-6.43 ± 0.23	-5.44 ± 0.36	4.53 ± 0.49
ΔGGAS	-34.26 ± 3.94	-27.62 ± 2.25	-53.46 ± 4.20	-45.84 ± 5.69	-32.53 ± 7.59
ΔGSOLV	18.79 ± 3.12	7.45 ± 1.95	23.17 ± 4.58	30.45 ± 4.31	21.19 ± 5.44
ΔTOTAL	-15.46 ± 2.78	-20.17 ± 2.77	-30.28 ± 3.42	-15.39 ± 2.91	-11.34 ± 5.59
